# 2-Keto-3-deoxy-D-xylonate and 2-oxo-4-hydroxybutyrate as natural and artificial effectors of transcription factors regulating D-xylonate operons in *E. coli*

**DOI:** 10.1007/s00253-026-13840-y

**Published:** 2026-05-01

**Authors:** Thibault Malfoy, Ceren Alkim, Julie Fredonnet, Juan Lajarin-Hernandez, Jean Marie Francois

**Affiliations:** 1 UMR INSA -CNRS5504 and UMR INSA-INRAE 792, Toulouse Biotechnology Institute, 135 Avenue de Rangueil, Toulouse, 31077 France; 2 Toulouse White Biotechnology, UMS INRAE-INSA-CNRS, 135 Avenue de Rangueil, Toulouse, 31077 France

**Keywords:** DNA-binding transcription regulator, D-Xylonate, 2-oxo-4-Hydroxybutyric acid, 2,4-Dihydroxybutyric acid, Synthetic pathways, Biosensor, Genetic circuits

## Abstract

In *E. coli*, the genes of the D-xylonate catabolic pathway are present in two distinct operons, *yagEFG* and *yjhIHG*, which are part of two cryptic phages found in the genome of this bacterium. These operons are under the control of two transcription factors: XynR which acts as a repressor of *yagEFG* and *yjhI* which is an activator of *yjhIHG*. Although D-xylonate is known to induce these two operons, the true inducer has not been identified yet. Through the construction of biosensors based on *xynR* and *yjhI*, using *syfp2* as reporter gene in combination with mutants of genes involved in D-xylonate catabolism, it has been demonstrated that the effector of these operons is 2-keto-3-deoxy-D-xylonate, which is formed by the dehydration of D-xylonate catalyzed by the dehydratases encoded by *yagF* and *yjhG* genes. Building on the finding that these two operons were also upregulated in *E. coli* challenged with high concentration of platform molecule 2,4-dihydroxybutyric acid, it is reported that both XynR- and YjhI-based biosensors were also responsive to the intermediate non-natural molecule 2-oxo-4-hydroxybutyric acid, with characteristic performances in terms of response threshold, sensitivity, cooperativity, and dynamic response comparable to those of the natural effector. Given that the bottlenecks in the production of 2,4-dihydroxybutyrate from C2 and C5/C6 carbon are D-threonate dehydratase and L-homoserine transaminase, respectively, which catalyze the formation of 2-oxo-4-hydroxybutyric acid from either D-threonate or homoserine, we showed that XynR and YjhI-based biosensors could be efficient tools for the screening and selection of more active enzymes producing this compound, thereby improving the production of 2,4-dihydroxybutyrate.

**Supplementary Information:**

The online version contains supplementary material available at 10.1007/s00253-026-13840-y.

## Introduction

**Fig. 1:**
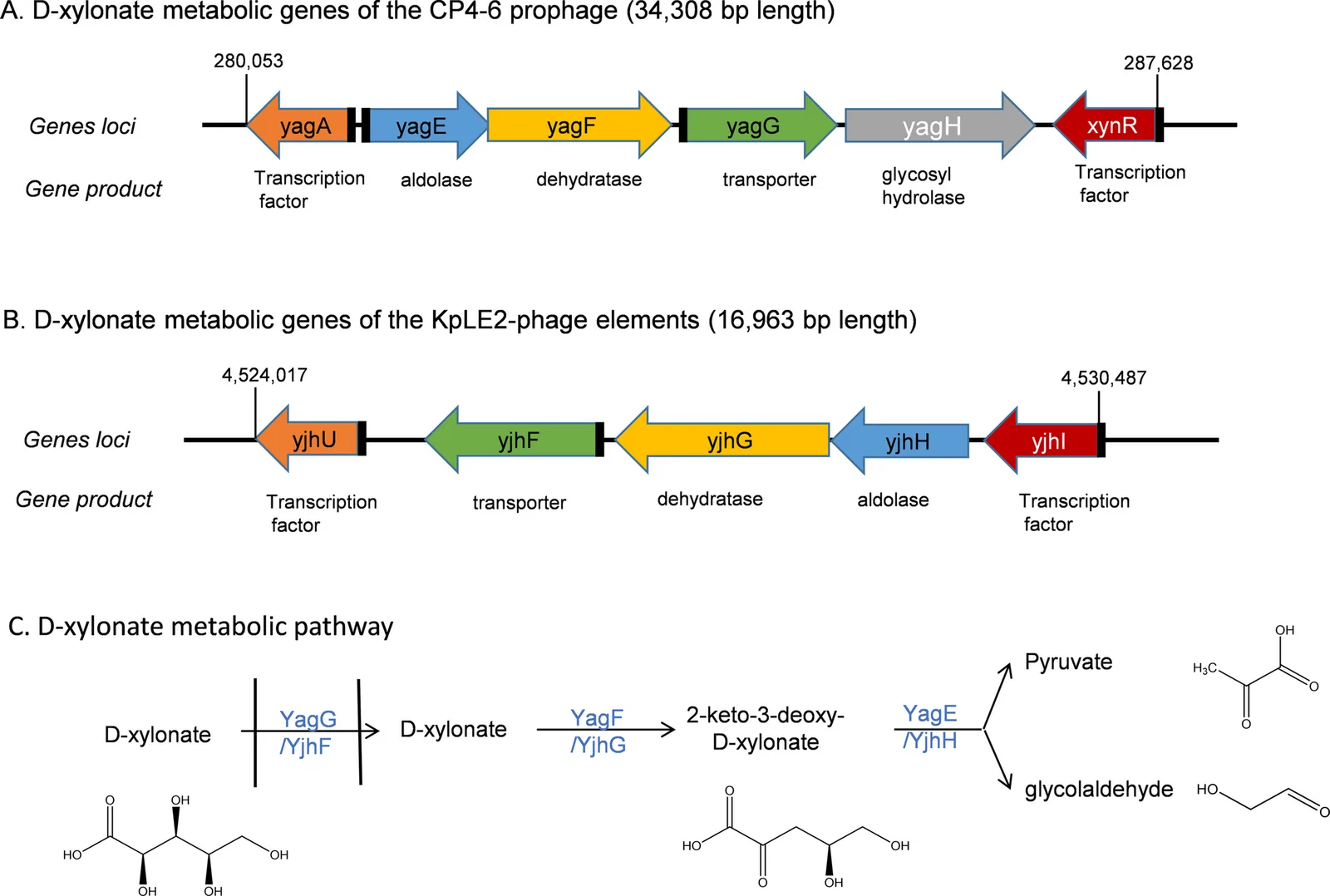
Scheme of the D-xylonate metabolic genes under XynR or YjhI transcription factor- regulation. **A** D-Xylonate metabolic genes in the 34,308 bp genomic structure of the CP4-6 cryptic prophage that are under the control of DNA binding transcriptional repressor encoded by *xynR*. **B** D-Xylonate metabolic genes in the 19,963 KplE2-phage elements that are under the DNA binding transcriptional activator encoded by *yjhI*. **C** D-Xylonate catabolic pathway

The Dahms pathway enables the assimilation of xylose into glycolaldehyde and pyruvate involving four enzymatic steps (Dahms 1974). The first reaction in this pathway is the oxidation of D-xylose to D-xylonate by a xylose dehydrogenase. While this enzyme does not exist in *E. coli*, the remaining enzymatic steps are encoded by two distinct operons, namely *yagEFGH* and *yjhIGHF* that are present on CP4-6 and KpL12-prophages, respectively, in the genome of this bacterium. Genetic and biochemical data have shown that *yagF* and *yihG* encode a xylonate dehydratase that converts D-xylonate to 2-keto-3-deoxy-D-xylonate (KDX). This enzymatic step is followed by an aldolic cleavage of KDX into glycolaldehyde and pyruvate by a pyruvate-dependent aldolase encoded by *yagE* and *yjhH*. D-Xylonate uptake is assumed to be taken up by a transporter encoded by *yagG* and *yjhF* (reviewed in Banares et al. 2021 and Fig. 1). It has been reported that these two operons are induced by D-xylonate through the action of a transcription factor. Shimada et al. (2017) showed that YagI, renamed XynR, which is also encoded by the CP4-6 prophage acts as a repressor of the *yag* operon, whereas Banares et al*.* (2019) demonstrated that YjhI, which is present on KpL12-phages-like elements, very likely works as an activator of the *yjh* operon, which also includes its autoinduction. However, it remained elusive whether D-xylonate is the direct effector of these two transcription factors. Two types of experimental data suggested that the effector of XynR and YjhI is likely not D-xylonate. On the one hand, the concentration of D-xylonate required to release the binding of XynR to DNA or to activate YjhI-dependent genes is in the range of 10 to 20 mM (Banares et al. 2019; Shimada et al. 2017), which is very high with respect to micro to millimolar concentration of most of metabolite effectors (Hanko et al. 2020). On the other hand, transcriptomic analyses of *E. coli* challenged with the non-natural platform molecule 2,4-dihydroxybutyric acid (2,4-DHB) triggered a strong upregulation of the D-xylonate catabolic genes (our unpublished data). Since metabolic pathways designed to produce 2,4-DHB are quite different to the catabolic pathway of D-xylonate, these data suggested that the effector of XynR and YjhI could be a metabolite derived from D-xylonate and that this metabolite may be structurally close to 2,4-DHB or its proximal intermediate 2-oxo-4-hydroxybutyrate (OHB).

To answer these questions, we designed a biosensor consisting of a reporter gene encoding a fluorescent protein dependent on the promoter of a gene under the control of XynR or YjhI. With this tool, and combined with mutants defective for D-xylonate catabolic pathway, we could on the one hand investigate the true effector of these two transcription factors and on the other hand, examine whether these TFs are responsive to 2,4-DHB or an intermediate in the production pathway of this molecule, in order to repurpose them as biosensors tool for improving the flux production of this non-natural platform molecule. The motivation for developing such biosensors sensitive to either 2,4-DHB or its proximal intermediate, 2-oxo-4-hydroxybutyrate (OHB), stems from the still low performance in terms of yield and productivity of the *E. coli* strains equipped with synthetic pathways designed for the bioproduction of this platform molecule from sugars or from C1/C2 carbon source (see Figure S1). Enzymes in these non-native pathways have been engineered to catalyze reactions on non-natural substrates, leading to the non-natural intermediate OHB, which is produced either from L-homoserine by the action of promiscuous transaminases (Bouzon et al. 2017; Walther et al. 2018) or from D-threonate by D-threonate dehydratase (Frazão et al. 2023). Clearly, these enzymes are rate limiting in the production pathways of 2,4-DHB because they harbor a catalytic efficiency (k_cat_/K_M_) at least 100-fold lower than most of the other enzymes needed for the bioproduction. Thus, directed enzyme evolution is required to optimize their catalytic efficiency, which relies on a high throughput screening method. Transcription factor-based metabolite biosensors would be well appropriate as long as OHB or 2,4-DHB can be recognized as a target of a transcription factor already existing in *E. coli*. Since nature has developed a wide range of sensors using natural endogenous metabolites that enable bacteria to adapt to their environment, the use of TF-based biosensors becomes challenging when it comes to optimizing a purely synthetic pathway whose intermediates and/or end product are completely unknown to the microorganism.

In this work, we not solely reported that the natural effector, and therefore the potential ligand, of XynR and YjhI is the intermediate metabolite KDX, but we also validated that both transcription factors can be repurposed as biosensors to be used for screening enzymes variants aiming to improve the flux production of the non-natural platform molecule 2,4-DHB.

## Material and methods

### Chemicals and reagents

All chemicals and solvents were purchased from Sigma-Aldrich unless otherwise stated. The 2,4–dihydroxybutyric acid (racemic form, ammonium salt) was a kind gift from Adisseo and was > 95% pure as determined by HPLC. The 2-oxo-4-hydroxybutyrate (OHB) was synthesized by incubating 125 mM D-homoserine with 4.5 U/mL porcine kidney D-amino acid oxidase, 4000 U/mL beef liver catalase in 100 mM Tris-HCl buffer (pH 8) for at least 2 h at 37 °C. These enzymes were purchased from Sigma-Aldrich. The reaction was filtered through AmiconTM Ultra filters (10 kDa threshold, Millipore). OHB was quantified by NMR using a Bruker Avance III HD 800 MHz spectrometer equipped with a cryogenically cooled 5 mm QCI-P (H/P-C/N/D) quadruple resonance probe. The spectra were acquired and reprocessed with Bruker Topspin 4.1 software, and were calibrated for reference to the frequency of TSP (TrimethylSilylPropanoic acid) on the 1H spectrum at 0 ppm. The solution, giving a yield of conversion of D-homoserine ranging from 77 to 88%, was stored at − 80 °C in small aliquots, which were used only once after thawing. The compound 2-keto-3-deoxy-D-xylonic acid (KDX) was purchased from Biosynth Ltd. and was reanalyzed by HPLC and ^1^H-NMR (see Figure S2) for its quality and purity which was roughly estimated at 90%.

Restriction endonucleases, Phusion polymerase, restriction enzymes, HiT4 DNA ligase, and HiFi DNA assembly were purchased from New England Biolabs and used according to instructions of the manufacturer. Moreover, In-Fusion® HD Cloning Kit was purchased from TaKaRa-Clontech. DNA plasmid isolation was performed using GeneJET Plasmid Miniprep Kit (Thermo Scientific). DNA extraction from agarose gel was carried out using the GeneJET Gel Extraction Kit (Thermo Scientific). DNA sequencing was carried out by Eurofins SAS (Ebersberg, Germany).

### Strains construction and growth conditions

List of strains and plasmids constructed and used in this work is reported in Table S1. The strain *E. coli* K-12 substr. MG1655 (ATCC 47076) was used as the parental strain for all constructions in this work. The strain MGΔ7 was constructed by deleting *eda, yfaU, garL, yagE, yjjH*, and *dgoA* encoding pyruvate-dependent aldolase in the *E. coli* genome by the phage transduction method adapted from Miller (Miller 1992), whereas a seventh gene, *mhpE*, was deleted using CRISPR-Cas9 method developed by Jiang et al. (2015a). In transduction procedure, donor strains were taken from the Keio collection (Baba et al. 2006), which contains mutants where a single gene is deleted and replaced by a kanamycin resistance cassette flanked by FRT sites. This method allowed the construction of simple, double and etc. mutants since all target genes were separated by at least 100 kb. Likewise, MG1655 was deleted of its membrane-associated lactate oxidoreductase encoded by *lldD*, *dld,* and *ykfEGF* by transduction giving rise to MGΔLO, as described elsewhere (Malfoy et al. 2024). Positive clones were selected on LB agar plates containing kanamycin (50 µg mL^−1^) and verified by PCR analysis. The Kan cassette was removed from the genome by expressing FLP recombinase from the pCP20 plasmid (Cherepanov and Wackernagel 1995) and correct excision of the cassette was verified by PCR using locus specific primers (Table S3). Other strain deletions, such as MG1655 *∆xynR*, MG1655 *∆yjhI*, and MG1655 *∆xynR ∆yjhI*, as reported in Table S1 were also carried out by P1 phage transduction according to (Miller 1992). Strain MGΔ4 defective in the aspartate-homoserine pathway (*∆thrA*, *∆metL*, *∆asd, ∆lysC*) was created as reported elsewhere (Malfoy et al. 2024). To obtain the mutant strain MGΔT which was deleted for four transaminases (*alaC*, *tyrB*, *ybdL*, *ilvE*) and *asd* encoding the aspartate semi-aldehyde dehydrogenase, the transaminases genes were consecutively deleted in MG1655 by transduction (Thomason et al. 2007). Then, *asd* gene was deleted in the obtained mutant MG1655 *∆alaC::FRT ∆tyrB::FRT ∆ybdL::FRT ∆ilvE::FRT* by the CRISPR-Cas9 method. This mutant was first transformed with pCas9. Guide RNA was expressed from a pTargetF-asd. Overnight cultures grown at 30 °C were diluted to an OD_600_ of 0.05 in LB containing kanamycin and arabinose (10 mM) and grown to OD_600_ ≈ 0.6. Cells were chilled on ice for 15 min, harvested at 5000 × *g* for 5 min at 4 °C, washed twice with ice-cold sterile water and once with 10% glycerol, and resuspended in 100 µL 10% glycerol. Competent cells were electroporated according to Wang et al. (2009) with 100 ng pTarget and 400 ng donor DNA (Eurofins Genomics) using a Gene Pulser Xcell (Bio-Rad) at 2.5 kV in a 2-mm cuvette. Cells were recovered in SOC medium for 1–2 h at 30 °C and plated on LB agar containing kanamycin and spectinomycin. Genome edits were verified by PCR and sequencing, and pTarget and pCas9 were sequentially cured from positive clones.

Unless otherwise stated, *E. coli* strains were cultivated in Lysogeny Broth (LB) medium at 37 °C on a rotary shaker running at 200 rpm. The antibiotics kanamycin, spectinomycin, or chloramphenicol were added when required at concentrations of 50, 100, and 25 mg L^−1^, respectively.

### Construction of the metabolite-based transcription factor biosensors

Plasmids constructed and used in this study are reported in Table S2 and primers needed for these constructions are reported in Table S3. Scheme of the constructed biosensor plasmids are reported in Figure S2. Construction of the various plasmid was initiated by generating pBS1 (not shown in Table S2) by inserting a 200 bp fragment starting at - 200 bp to - 1 bp before ATG codon of *yagE* that was amplified from MG1655 genomic DNA using primers TM7 and TM8, adding *XhoI* and *HindIII* restriction sites on 5' and 3' ends. The PCR product was ligated with HiT4 DNA ligase into gel purified XhoI-HindIII digested pREP22 vector from (Frazao et al. 2018) that carries *syfp2* gene encoding the super yellow fluorescent protein (Kremers et al. 2006), which also carries the optimized RBS already present on the pREP22 plasmid between the promoting region of *yagE* and the *syfp2* gene. The pBS2 was then obtained from pBS1 as follows: the *xynR* gene was inserted into pBS1 using a 759 bp corresponding to the coding sequence of this gene along with 190 bp upstream the ATG codon. This fragment was obtained from MG1655 strain by PCR amplification using primers TM4 and TM11. Meanwhile, T7 terminator was amplified from pET-28a with TM5 and TM14. These primers allowed insertion of flanking regions corresponding to linearized pBS1 carried out with TM12 and TM13 primers. These three fragments were gel purified and incubated with HiFi DNA assembly mix to generate pBS2 plasmid, which was validated by sequencing after amplification in NEB-5α competent *E. coli* cells and purification. To generate pBS3, primers TM71 and TM146 were used to amplify the *syfp2-xynR* cassette of pBS2 plasmid whereas medium copy vector pZA33 was amplified with primers JFR64 and JFR65. Both fragments were gel-purified and ligated using NEBuilder HiFi DNA assembly. Validation of pBS3 was done by sequencing using primers JFR66/JF67 and TM10/TM16. To obtain pBS5, *lldD* gene from the genome of *E. coli* MG1655 was amplified with primers TM67/68, λ tL3 terminator of Cas9 was amplified from pCas plasmid (Jiang et al. 2015a) using primers TM69 and TM70 and pBS2 using primers TM71 and TM72. All these fragments were gel purified and assembled with HiFi DNA assembly, to form pBS5. In addition, a DNA fragment that contains pJ23106 promoter (ordered from Eurofins genomics, France) and Shine-Dalgarno sequence was obtained by mixing primers TM63 and TM64 at 70 °C, followed by slow decrease of temperature and then amplified with primers TM65 and TM66. After purification of the different fragments on agarose gel, they were assembled by the NEBuilder HiFi DNA Assembly (Biolabs) to generate pBS5, which was validated by sequencing using primers TM9/TM10/TM15/TM16 after amplification in NEB-5α competent *E. coli* cells and purification. Plasmid pBS10 was constructed from pBS5 by replacing the *lldD* gene with *yagE*. The *yagE* gene was amplified from the *E. coli* genome using primers 3173 and 3174. In parallel, the pBS5 plasmid was linearized by PCR using primers 3168 and 3175, which also resulted in the removal of the *lldD* gene. The amplified *yagE* fragment was then inserted into the linearized plasmid using an In-Fusion assembly reaction.

In-Fusion assembly protocol was also used to construct pBS7, pBS8, pBS6, and pBS9 (see scheme of these plasmids in Figure S2 in supplementary material). Whenever plasmids were used as templates for PCR amplification, the PCR products were treated with DpnI (New England Biolabs) to eliminate the template plasmid. Plasmid pBS3 was used as the template to generate pBS0, pBS6, and pBS9. Biosensor pBS0 was obtained by first removing the transcriptional factor *xynR* and the reporter gene *syfp2* with the *yagE* promoter from pBS3 by PCR (primers 3135 + 3136) and then reinsertion of the SYFP2 cassette as follows. The *syfp2* gene with the *yagE* promoter and its corresponding T7 terminator was amplified by PCR (primers 3131 + 3132). After PCR purification, a second amplification was performed to create 15 bp homology regions with the first template DNA. Following the In-Fusion assembly reaction, each plasmid construct was transformed into competent *E. coli* NEB-5α cells according to the manufacturer’s instructions. Transformed cells were plated on LB agar supplemented with chloramphenicol (25 µg/mL final concentration). Colonies were initially screened by PCR, and plasmids were extracted from positive clones. Two confirmed constructs were sent for full plasmid sequencing (Eurofins Genomics). These procedures were repeated whenever new plasmid constructs were obtained. Plasmid pBS6 was obtained by replacing the transcriptional factor XynR with YjhI in pBS3. For this purpose, chromosomal DNA from *E. coli* MG1655 was used to amplify *yjhI*, including 199 bp upstream of the ORF to cover its native promoter. A T7 terminator was added 49 bp downstream of *yjhI* during the same PCR reaction (primers 3130 + 3137). The purified product was then subjected to a second PCR to add 15 bp homology regions (primers 3128 + 3129). In parallel, pBS3 was used to amplify the plasmid backbone lacking *xynR* and its promoter region (primers 3126 + 3127). The plasmid backbone and the amplified *yjhI* fragment were assembled using the In-Fusion reaction to generate biosensor pBS6. Plasmids pBS8 and pBS9 were constructed using the same approach, starting from pBS6 and pBS3, respectively. In pBS6, the *yagE* promoter of the reporter gene was replaced with the *yjhI* promoter to generate pBS8. Similarly, replacement of the *yagE* promoter with the *yjhI* promoter in pBS3 yielded pBS9. Finally, pBS7 was constructed by overlap PCR using pBS8 as the template and overlapping primers 3154 + 3155, which removed the transcriptional factor *yjhI* and its promoter. The primer 3154 can anneal to both the upstream and downstream regions of P_*yjhl*_ + *yjhI* and its corresponding T7 terminator, respectively, in order to eliminate P_*jjhI*_ + *yjhI*. The reverse primer 3155 was designed to contain a 50-bp homology region corresponding to the terminal sequence of primer 3154. PCR was performed using pBS8 as the template with these primers. Following DpnI treatment and PCR clean-up, the product was transformed into *E. coli* NEB-5α cells. After PCR verification, positive clones were sent for whole-plasmid sequencing. The correct plasmids were kept at – 20 °C for further experiments.

For the construction of pBios-JF2 through JF6, pBS3 was used as the backbone. The primers seq-R and seq-F were used on pBS3 to yield after a DNA fragment without the promoter sequence of *xynR*. Then, the insertion of new promoter sequences bearing a strong RBS_01_ calculated according to Salis (2011) was carried out by assembling this linearized fragment with PCR amplified synthetic fragments obtained with primer seq-xynR_F on the 5’ end and one of the 5 other primers (Table S3) on 3’-end using the NEBuilder HiFi DNA Assembly (Biolabs). All the constructs were validated by sequencing.

### Construction of plasmids for screening assays of OHB producer enzymes

Removal of *aspC* in plasmid pZS2-aspC-kdgT-*Hh.*araD (Frazão et al. 2023) was done by reverse PCR using primers TM250 & TM251. In the resulting plasmid, pZS2-kdgT-*Hh.*araD, remove *araD* was removed by reverse PCR using primers TM252 & TM253 to yield pZS2-kdgT*.* The T7-inducible promoter of *alaC* gene and its mutant alaC**, encoding the variant AlaC^A142P Y275D^ (Bouzon et al. 2017), initially cloned in pET28a were replaced by T5 promoter to allow IPTG induction in MG1655 strain. To this end, pET28a was amplified using the primers TM233 and TM234, while T5 promoter was amplified from the PCA24N vector from the ASKA library (Kitagawa et al. 2005) using primers TM242 and TM243. Both fragments displayed a floating tail of 10 nt on each side that are homologous to the ends of the other amplicon. The T5 promoter was cloned using the HiFi DNA Assembly Master Mix from NEB, following the manufacturer guidelines. The resulting vector was named pETM28, carrying the T5 promoter upstream of the coding sequence. Wild-type *alaC* and mutant alaC** were amplified using the primers 917, bearing a *Sac*I site and 926, bearing a *Hind*III site. pET28M and the inserts were digested with *Sac*I and *Hind*III, gel purified and ligated using the HiT4 DNA ligase (NEB), following the supplier recommendations. The constructed plasmids pET28M_alaC and pET28M_alaC** were validated by sequencing.

### In vivo fluorescence experiments

Unless otherwise stated, bacterial culture for in vivo fluorescence experiments were performed as follows. At day 1, a preculture of the bacterial strain was carried out in 2 to 5 ml LB medium with appropriate antibiotic in 50-ml falcon tube at 37 °C in rotary shaker set at 200 rpm. The next day, the cultures were diluted in 5 ml of either LB or in M9/MOPS mineral medium containing 4 g/L xylose with the appropriate antibiotics in 50 ml falcon tubes at an initial OD_600_ of 0.05. After 3 h and half for LB or 6 h for M9/MOPS, the cultures were collected by centrifugation at 3250*xg* for 15 min at room temperature, washed once with 5 ml PBS buffer, centrifugated again, and then resuspended, unless otherwise stated, in 5 ml of PBS. Three hundred µl of the culture were delivered in 48-well microtiter plates and each culture was made in triplicate with independent bacterial clones. After 5-min incubation at 37 °C, metabolites/effectors as indicated in corresponding figures were added. Growth at 600 nm and fluorescence of the SYFP2 protein were measured over a time period of 16 h either using a spectrofluorometer Clario Star from GmbH Labtech (excitation and emission wavelength set at 515 nm and 527 nm, respectively) or with a Biotek Synergy HTX from Agilent/Thermo Scientific using excitation filter at 485 and emission filter at 525 nm. The fluorescence expressed in arbitrary unit (AU) corresponds to the absolute fluorescence divided by OD measured at 600 nm. Fluorescence was also determined by flow cytometry using a BD Accuri™ C6 Plus Personal Flow Cytometer (BD Biosciences) with excitation set at 488 nm. Forward-scatter characteristics (FSC) and side-scatter characteristics (SSC) were detected as small-angle and large-angle scatters of the 488-nm laser, respectively. SYFP2 fluorescence was detected using a 530/30 nm (channel FL1) band-pass filter set. A total of 100,000 events were recorded per sample, and electronic gating was applied on the densest subset of cells based on forward- versus side-scatter height. The same gate was used to estimate median levels of SYFP2 fluorescence.

The protocol was slightly modified for the screen of transaminases as the gene encoding these enzymes were cloned in a plasmid requiring their induction by IPTG. Therefore, after overnight culture in LB with appropriate antibiotics, the cells were reinoculated in LB with the appropriate antibiotic at OD_600_ 0.05, cultivated for another 2 h, after which 0.5 mM IPTG was added and growth in an incubator set at 37 °C and 200 rpm was continued for 4 h. Cells were collected by centrifugation, washed once with PBS, and resuspended in PBS as above. For threonate dehydratase, no IPTG was added since the expression of the gene was constitutive, and after overnight culture in LB, the culture was washed twice with PBS, resuspended in PBS at OD_600_ ≈ 1.0 to which was added D-threonate (10 mM), OHB (1 mM), or water. The fluorescence/growth was monitored as described above using the Biotek Synergy HTX fluorometer or by flow cytometer.

### Data processing and statistical analysis

For each data sets, absolute fluorescence intensity and OD_600_ were recorded and the relative fluorescence in AU was obtained by dividing absolute value to OD. All experiments were done at least with three biological triplicates. Excel tools were used to calculate the mean, standard deviation and covariance (CV). Determination of the response threshold of the biosensor to its metabolites was obtained by fitting a nonlinear regression curve of the fluorescence intensity versus the concentration of the substrate using Solver tool in Excel or Graph Pad Prism V10.6.1.

## Results

### Construction of the XynR and YjhI-based biosensor and investigation of their responses to D-xylonate

**Fig. 2:**
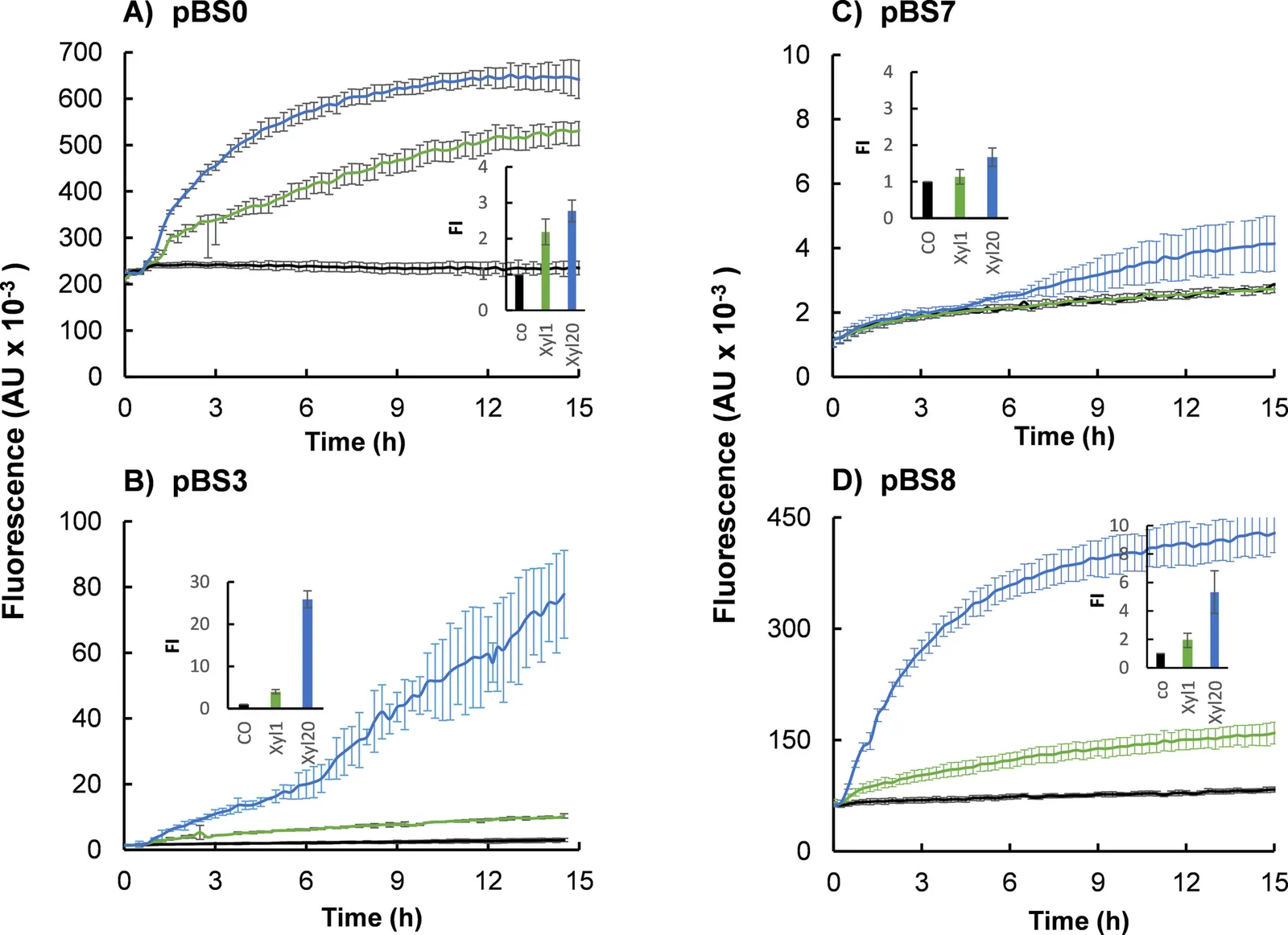
Time course response of XynR and YjhI-based biosensors to D-xylonate. *E. coli* MG1655 strain was transformed with pBS0, pBS3, pBS7, or pBS8 (see Table S2 & Figure S3 for description of these plasmids). The fluorescence emitted by SYFP2 protein after addition of 1 mM or 20 mM D-xylonate (Xyl1, Xyl20, co means no addition) was recorded over 15 h in spectrofluorometer together with OD_600_ as described in the “Material and methods” section. Fluorescence intensity was normalized to OD, given arbitrary units (AU). The fold induction (FI) by D-xylonate at 12 h of incubation was calculated by dividing the fluorescence (AU) in response to D-xylonate to that of the control. The data are the mean ± SD (shown by bars on the curves and histograms) of 3 biological replicates

According to previous works, the transcription factor encoded by *xynR* acts as a repressor of D-xylonate metabolic genes present in the cryptic CP4-6 prophage whereas YjhI transcription factor is an activator of similar genes that belong to the KpLE12 phage-like elements (Fig. 1). To confirm these data, we build four types of biosensors on a medium copy plasmid, using *syfp2* encoding the super yellow fluorescent protein as the readout (Kremers et al. 2006). For the two first biosensors carried on pBS0 and pBS7, *syfp2* was under the control of a XynR or YjhI-dependent promoter, while the two others plasmids in addition carried *xynR* (pBS3) and *yjhI* (pBS8) driven by their own promoter (see Figure S3 in Supplementary data). These plasmids were inserted by transformation into *E. coli* MG1655 strain, and fluorescence was measured over time in response to D-xylonate added at 1 or 20 mM. As shown in Fig. 2, the addition of this sugar acid triggered an increase in fluorescence intensity with both pBS0 and pBS3. However, the basal fluorescence in pBS3 was 200 times lower than that measured with pBS0, and the response time at both D-xylonate concentration was faster with pBS0 than with pBS3. These data are consistent with the repressive effect exerted by XynR, present in greater quantities in strain carrying pBS3 than pBS0 since in the latter, only *xynR* is expressed from its genomic copy. Unlike XynR, the addition of D-xylonate at 1 or 20 mM caused a weak increase of fluorescence in strain transformed with pBS7, while the basal fluorescence in strain transformed with pBS8 was about 50-fold higher than in strain carrying pBS7. These results suggested that the expression of *yjhI* from its chromosomal locus in strain carrying pBS7-is insufficient to produce enough protein capable of activating *syfp2*, what is achieved thanks to the increase in the number of copies of this gene when it is placed on a medium copy plasmid (pBS8). In conclusion, the XynR-based biosensor harbors the typical repressed-repressor architecture whereas the activated-activator architecture characterizes the YjhI-based biosensor (Mannan et al. 2017).

### The metabolite effector of XynR and YjhI is the intermediate 2-keto-3-deoxy-D-xylonate (KDX)

**Fig. 3:**
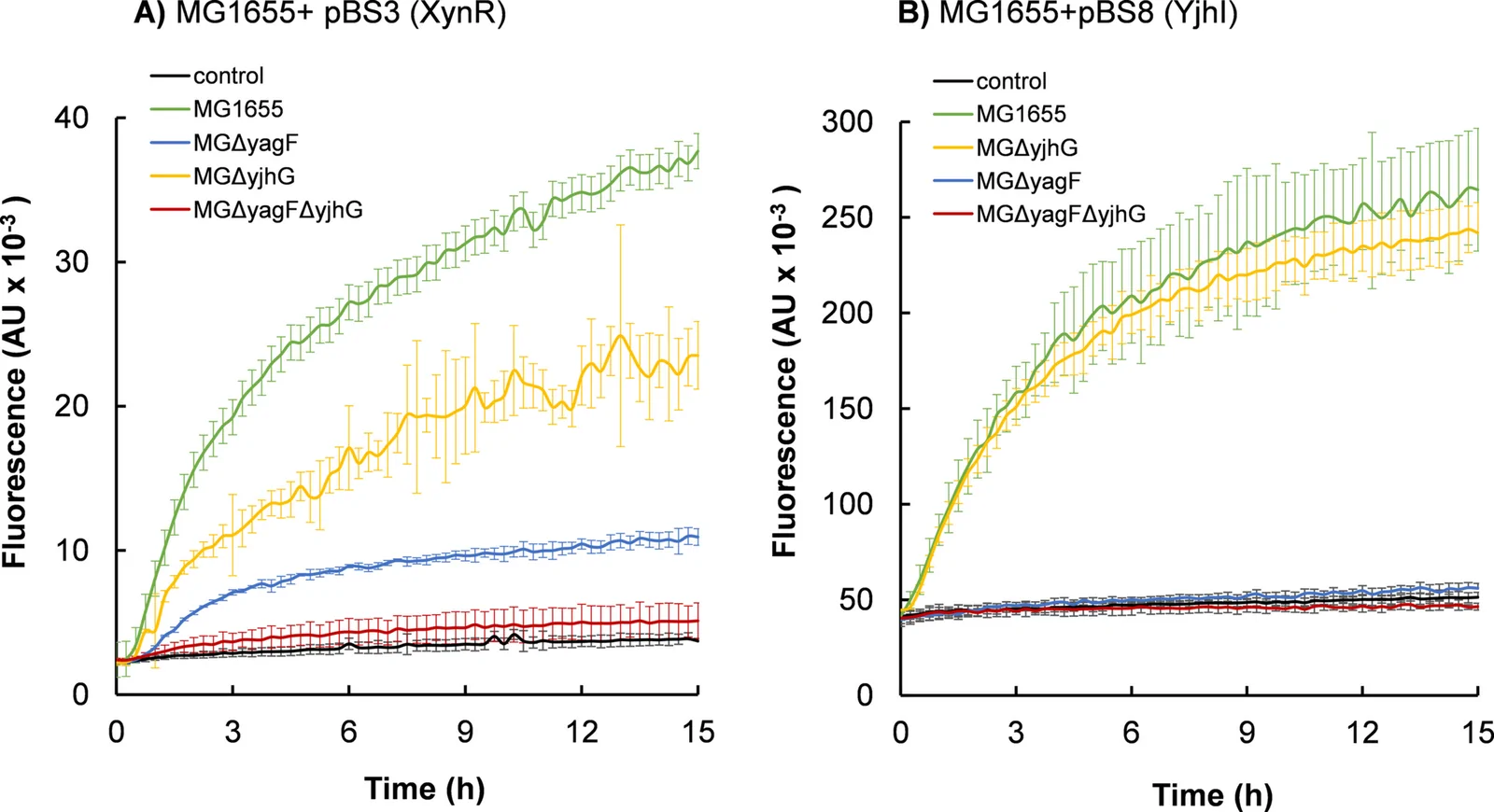
Lack of response of XynR and YjhI-based biosensors to D-xylonate in mutants defective in D-xylonate dehydratase. Same experiment as in Fig. 2 except that *E. coli* MG1655 and mutant defective in the D-xylonate dehydratase encoded by *yagF* and *yjhG* were transformed with pBS3 or pBS8. The concentration of D-xylonate was 20 mM. The data reported are the mean ± SD (shown by bar of the curves) of 3 biological replicates

D-xylonate is known to induce XynR and YjhI-regulated operons but whether this sugar acid was the direct effector sensed by these TFs remained an open question (Banares et al. 2019; Shimada et al. 2017). The use of the XynR and YjhI-based biosensors developed above could be useful to address this question, notably in light with the observation that the fluorescence kinetics in response to D-xylonate was preceded by a lag period of few minutes to one hour (Fig. 2). This lag period could be due either to the time needed to release all XynR-molecules that bind to *yagE* promoter, to the time for sufficient autoactivation of YjhI or to the time required to produce and accumulate a metabolite from D-xylonate. To solve this issue, we deleted *yagF* and *yjhG* which code for a D-xylonate dehydratase that catalyzes the dehydration of D-xylonate into 2-keto-3-deoxy-D-xylonate (KDX) (see Fig. 1). Remarkably, XynR-and YjhI-based biosensors expressed in a mutant defective in both genes were no longer responsive to D-xylonate (Fig. 3). Furthermore, this experiment showed that the fluorescence signal in the *yagF* mutant expressing the XynR-based biosensor was weaker than in the *yjhG* mutant in response to D-xylonate, and that there was even no increase in the fluorescence signal in the *yagF* mutant transformed with the pBS8 vector in response to D-xylonate. These results suggested that the D-xylonate dehydratase activity is more dependent in vivo on the expression of *yagF* than *yjhG*. To further support the genetic evidences of KDX being the effector sensed by these transcription factors, MGΔ7 was transformed with pBS10, which carried, in addition to *xynR* and *syfp2*, *yagE* encoding a pyruvate-dependent aldolase of the D-xylonate pathway. As expected, given the catalytic property of this aldolase, which can reversibly produce KDX by the condensation of pyruvate and glycolaldehyde, an increase of fluorescence as expressed by fold induction was measured after the addition of these two metabolites together, whereas the separate addition of pyruvate and glycolaldehyde did not result in any significant increase in fluorescence as compared to control (Figure S4). One could notice that the fold induction in response to either KDX or OHB used as control was only threefold. This weak induction could be due to the fact that pBS10 is a high copy plasmid, leading to a higher expression of xynR and hence much higher amount of the repressor, with consequence on reduced dynamic response of the biosensor (Liu et al. 2015; Mannan et al. 2017).

**Fig. 4:**
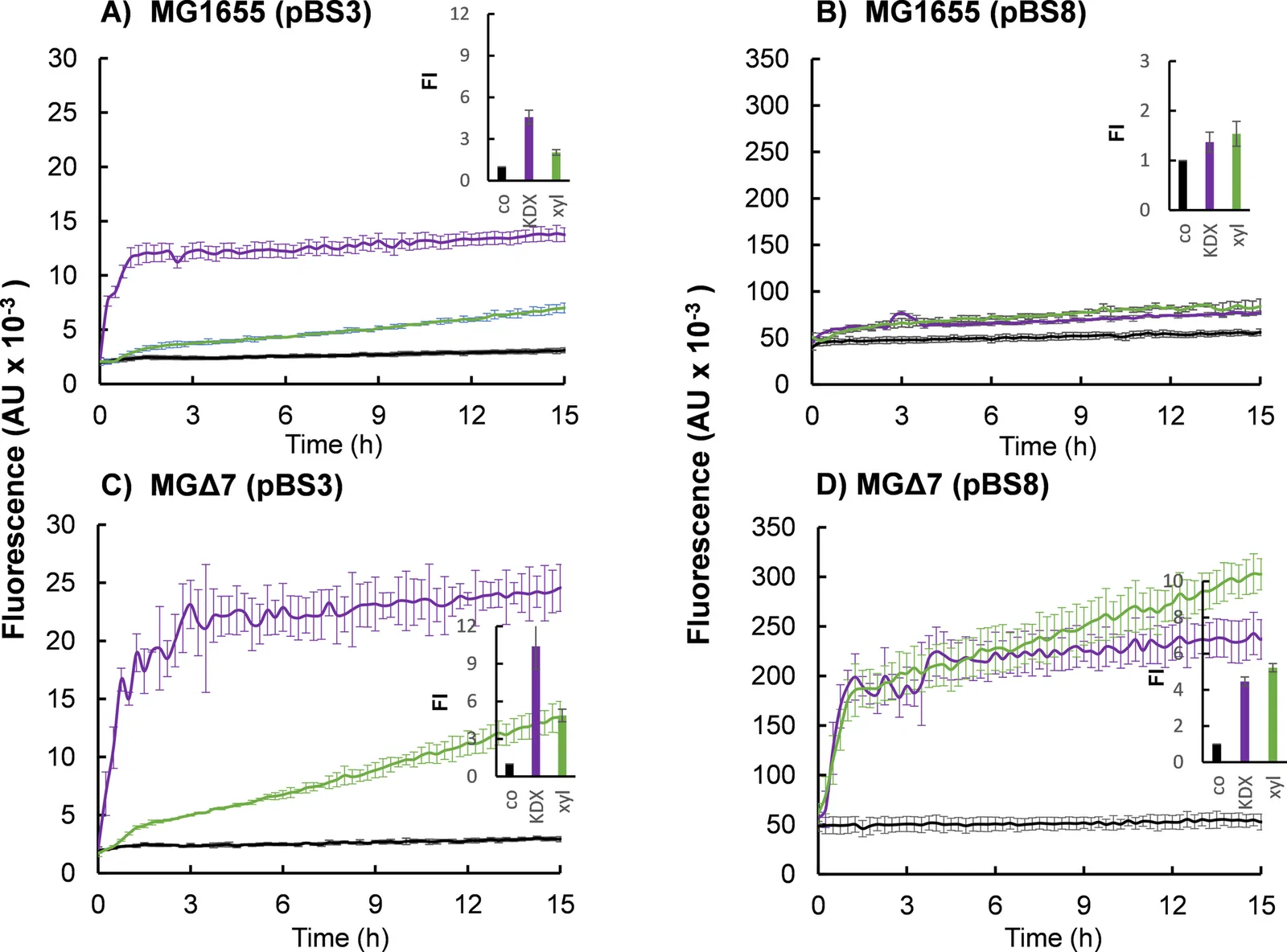
The intermediate 2-keto-3-deoxy-D-xylonate is the metabolite effector of XynR and YjhI transcription factors. Same procedure as in Fig. 2 except that wild-type MG1655 and mutant defective in major pyruvate-dependent aldolase (MGΔ7) was transformed with pBS3 or pBS8. D-xylonate (Xyl) and 2-keto-3-deoxy-D-xylonate (KDX) were used at 1 mM. The fold induction (FI) given at 12 h of incubation was calculated as in Fig. 2. Data are the mean ± SD (shown by bars on the curves and histogram) of three biological replicates

Based on these data, we investigated the effect of KDX as a potential metabolite effector of XynR and YjhI transcription factors. As shown in Fig. 4A**,** the addition of 1 mM KDX to MG1655 carrying XynR-based biosensor on pBS3 resulted in a fluorescence response that was faster and higher than after addition of 1 mM D-xylonate. Moreover, the increase in fluorescence was more potent in the mutant strain MGΔ7 which is deleted for *yagE* and *yjhH* encoding promiscuous aldolases reported to cleave KDX into pyruvate and glycolaldehyde (Bhaskar et al. 2011; Liu et al. 2013). With the YjhI-based biosensor carried by pBS8, the increase of fluorescence in response to KDX and 1 mM D-xylonate was very weak, but a four- to fivefold induction was measured in the aldolase-deficient MGΔ7 mutant. This net increase in fluorescence can be explained by the metabolization of D-xylonate into KDX which is no longer degraded in this mutant lacking all major KDX-cleaving aldolase (Fig. 4D). Altogether, these data support the notion that the direct effector of XynR and YjhI transcription factor is the intermediate KDX and not D-xylonate.

### The transcription factors XynR and YjhI are responsive to the non-natural molecule 2-oxo-4-hydroxybutyrate (OHB)

**Fig. 5:**
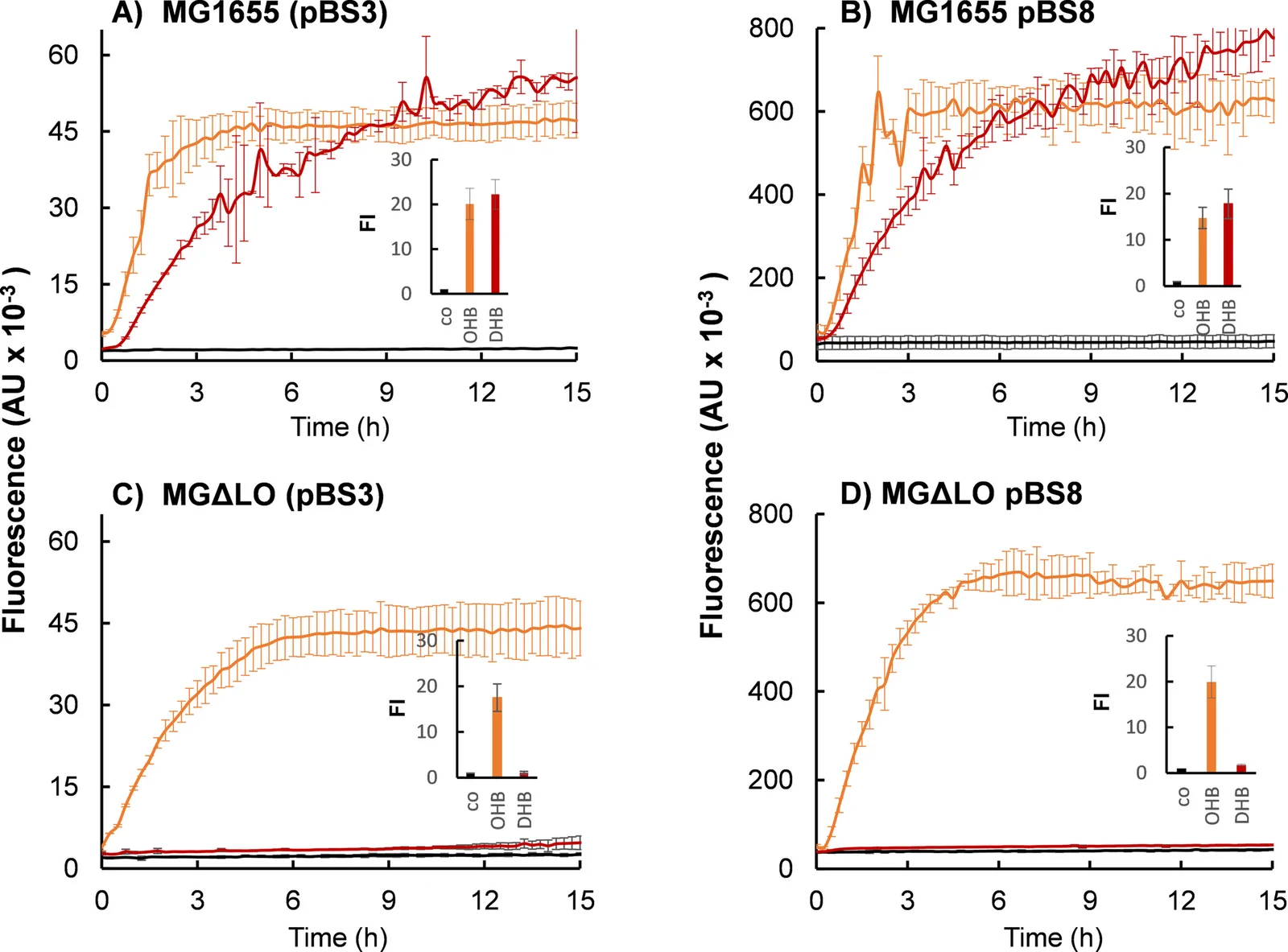
XynR and YjhI are responsive to 2-oxo-4-hydroxybutyrate and not to 2,4-dihydroxybutyrate. Same procedure as in Fig. 2 except that wild-type MG1655 and mutant defective in membrane-associated lactate dehydrogenase encoded by *lldD*, *dlD*, and *ykgEFG* (MGΔLO) was transformed with pBS3 and pBS8. 2,4-Dihydroxybutyrate (2,4-DHB) and 2-oxo-4-hydroxybutyrate (OHB) were added at 100 and 1 mM, respectively, and the control was done with 100 mM NH_4_Cl as NH_4_^+^ is the counterion of 2,4-DHB. The fold induction (FI) given at 12 h of incubation was calculated as in Fig. 2. Data are the mean ± SD (shown by bars on the curves and histogram) of three biological replicates

A transcriptomic analysis of an *E. coli* MG1655 engineered for the production of 2,4-DHB by fermentation from glucose (Walther et al. 2018) revealed a strong upregulation of D-xylonate catabolic genes from the CP4-6 and KpLE2 -phage elements during the production phase of this non-natural metabolite, with notably *yagE* and *yagF* being increased by 13.6- and 9.1-fold, respectively (see data in Table S4). Interestingly, *yjhI* was also upregulated by about eightfold, which was accompanied by an increased expression, albeit weak, of *yjhH* and *yjhG*. Based on these data, we asked whether 2,4-DHB or OHB could mimic the effect of KDX to activate the XynR- and/or YjhI-based biosensor. Results of this experiment are reported in Fig. 5. DHB was used at 100 mM because its action on the D-xylonate dependent operons was expected at high concentration whereas OHB was used at 1 mM because intracellular concentration of this metabolite was estimated in the millimolar range (our unpublished data). It can be seen that both 2,4-DHB and OHB caused a comparable and significant increase in fluorescence intensity over time with both biosensors. Also, the dynamic response expressed as fold induction was very similar for the two compounds on the two TFs-based biosensors. However, the time response to OHB was clearly faster than that of 2,4-DHB, suggesting that the effect of the latter molecule was not direct but could result from its metabolization. We recently reported that 2,4-DHB can be oxidized into OHB by membrane-associated lactate oxidoreductases encoded by *lldD*, *dld*, and *ykgEFG* (Malfoy et al. 2024). We therefore repeated this experiment using the MGΔLO strain deficient in these lactate oxidoreductases. As shown in Fig. 5C and D, the increase of fluorescence signal upon addition of 2,4-DHB was totally abolished in this mutant expressing the biosensor carried on pBS3 or pBS8. In contrast, the absence of lactate oxidoreductase had no effect on the response of these biosensors to OHB, indicating that this molecule is likely a direct effector of XynR and YjhI.

### No crosstalk in the response of XynR and YjhI to KDX and OHB

**Fig. 6:**
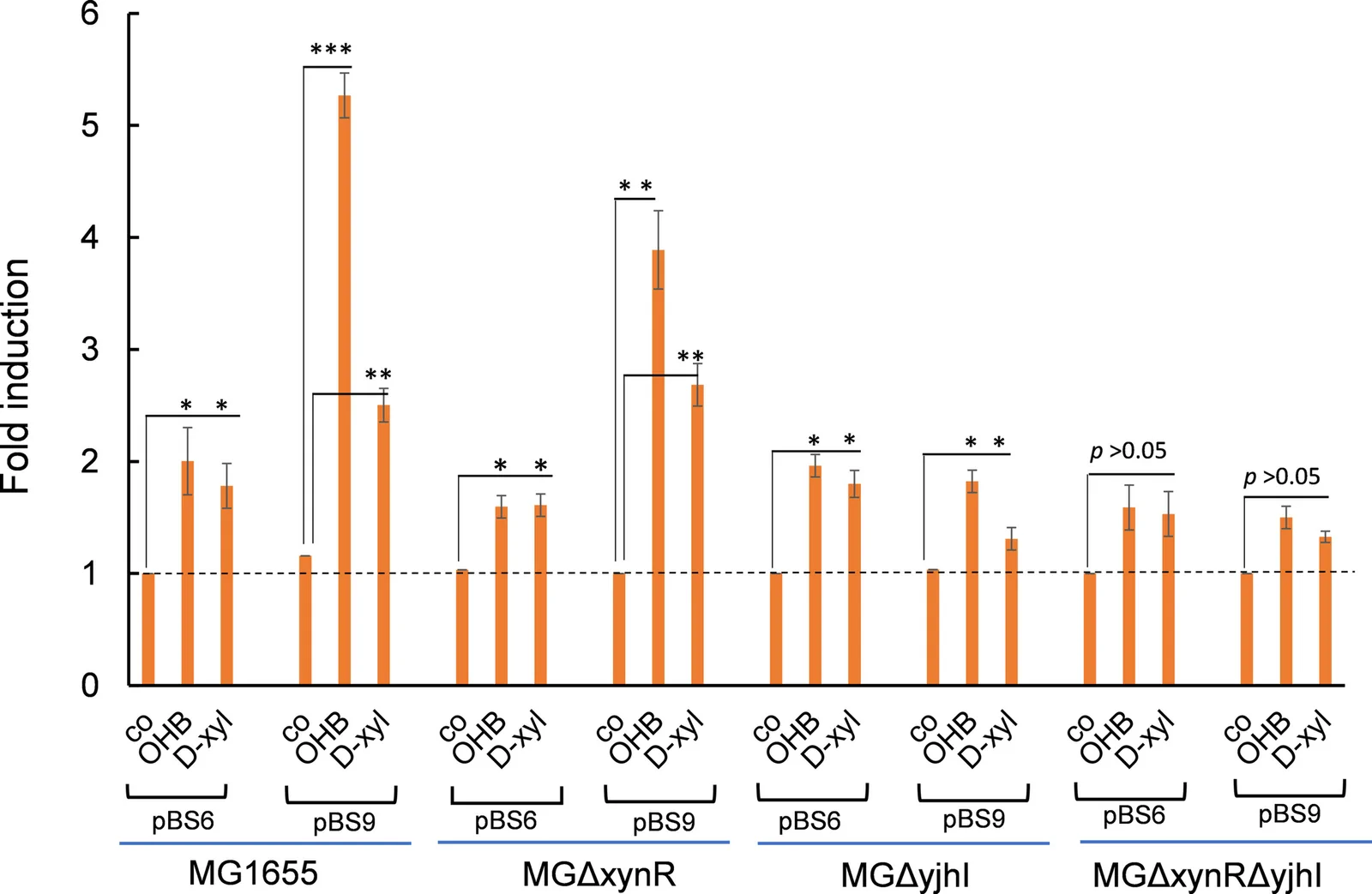
No cross-talk between XynR- and YjhI-based biosensors in response to OHB and D-xylonate. Same procedure as in Fig. 2 except that strain defective in either *xynR*, *yjhI*, or both *xynR* and *yjhI* were used together with the reference MG1655. Plasmids pBS6 and pBS9 (see description in Figure S3) were inserted in these strains by transformation. The response of the biosensors expressed from pBS6 and pBS9 were measured in the absence (co) or the presence of 1 mM OHB or 20 mM D-xylonate. The fold induction reported in the figure was determined from fluorescence after 12 h of incubation at 37 °C. Data are the mean ± SD (shown by bars on the curves and histogram) of three biological replicates. Statistical significance was determined by Student’s *t*-test (*p < 0.05; **p < 0.01; ***p < 0.001)

XynR and YjhI are predicted to belong to IclR-type transcription factor (Gao et al. 2018). However, neither the consensus binding sequence on the promoter of the regulated genes, nor the structure by which these TFs bind to DNA have been characterized yet. While it is currently admitted that activators generally bind upstream of the promoter while repressors preferentially bind downstream or overlapping the promoter, a same DNA binding transcription factor can act as either an activator or a repressor if it binds at different locations in the promoter (Dong and Guertin 2026; Rydenfelt et al. 2014; Tuğrul et al. 2015). To investigate such possible dual action or a possible crosstalk between XynR and YjhI in their regulatory function, we constructed plasmid pBS9 bearing *xynR* under its own promoter while *syfp2* reporter gene was under *yjhI* promoter and plasmid pBS6 that carried *yjhI* under its own promoter with *syfp2* under *yagE* promoter (see Figure S3 for the scheme of these constructions). The reference strain MG1655, single mutant, and double mutants defective in *xynR* and *yjhI* were transformed with these two plasmids and challenged with 1 mM OHB or 20 mM D-xylonate, which was used instead of KDX due to the limited availability of this compound. This experiment showed that the fluorescence induction in response to OHB and D-xylonate in MG*ΔxynRΔyjhI* mutant strain showed barely no significant difference as compared to the control (Fig. 6). This result supported the notion that each transcription factor controls only the genes of its own operon, and therefore indicated the absence of dual function and cross-talk in the control of these operons by D-xylonate. The 3 to sixfold induction observed in response to D-xylonate and OHB with pBS9 in both MG1655 and MGΔ*xynR* can be ascribed to the action of *yjhI* present in the genome, which triggered expression of *syfp2* gene reporter that is under the *yjhI* promoter in this plasmid. Conversely, the weak fold increase in response to OHB and D-xylonate in MG1655 or MGΔ*yjhI* strains carrying pBS6 was likely due to the low level of XynR protein, as the gene is expressed from the chromosomal copy, and hence, its low abundance resulted in an already high expression of *syfp2* gene that is weakly enhanced by addition of OHB or D-xylonate. This behavior resembled that found with MG1655 expressing pBS0 (see Fig. 2).

### Characteristic performances of the XynR and YjhI-based biosensor to KDX and OHB

**Fig. 7:**
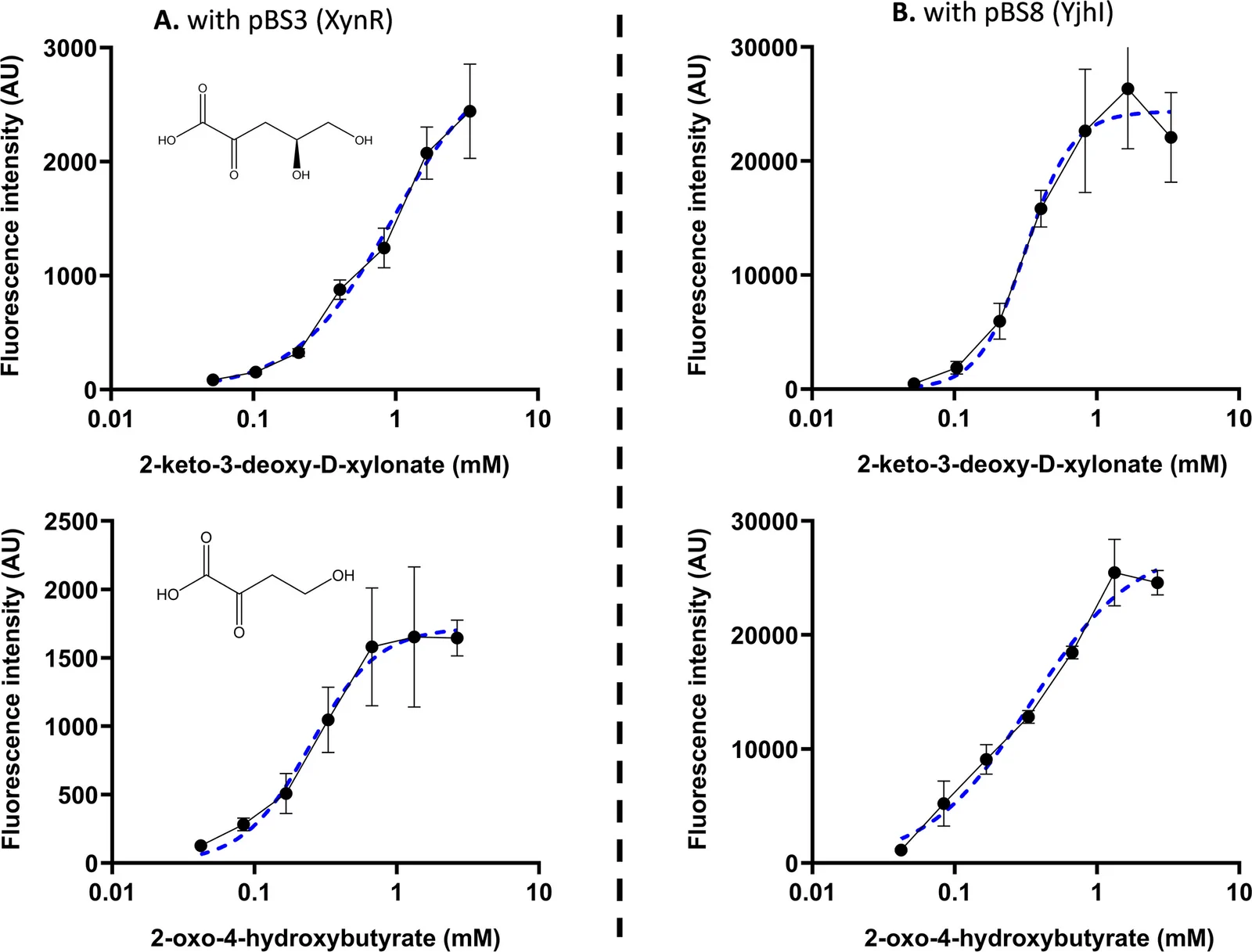
Dose–response curves of XynR and YjhI-based biosensors to 2-keto-3-deoxy-D-xylonate (KDX) and 2-oxo-4-hydroxybutyrate (OHB). The strain used for KDX and OHB dose–response was MGΔ7 transformed with pBS3 and pBS8. The fluorescence was measured by spectrofluorometer over 15 h and values at 12 h after addition of the effectors were used for dose–response analysis, using Graph Pad Prism for data treatment and graph representation. Data are the mean ± SD (shown by bars on the curves) of four biological replicates, each containing 2 technical replicates. The dotted blue lines were obtained from least squares regression smoothing with the Graph Pad Prism v10.2

As clearly outlined in the reviews by F. Zhang and colleagues (Hartline et al. 2021; Mannan et al. 2017), several criteria can be used to evaluate the biosensor performances which include the response threshold (i.e., K_0.5_), defined as the concentration of metabolite required for 50% of the maximal expression or induction, the detection range and the dynamic range corresponding to the maximal fold increase relative to the baseline. To evaluate these parameters for XynR-and YjhI-based sensors to OHB and KDX, MGΔ7 strain deleted for the so far seven identified genes encoding pyruvate-dependent aldolase (He et al. 2020) was used to prevent the metabolization of KDX into pyruvate and glycoladehyde (for KDX) and OHB into pyruvate and formaldehyde (Banares et al. 2021; Malfoy et al. 2024). Results reported in Fig. 7 showed that the XynR-based biosensor turned to be more sensitive to OHB than KDX (K_0.5_ ≈ 0.27 vs 1.0 mM, Table 1), while a comparable K_0.5_ in the range of 0.3–0.4 mM was obtained for both metabolites with the YjhI-based biosensor (Table 1). Furthermore, the cooperative behavior of the two TF-based biosensors expressed by the Hill number (n^H^) was significantly different between KDX and OHB. An apparent positive cooperativity of the XynR-based biosensor with OHB (n^H^ ≈ 2.05) was determined which was not observed with KDX. The opposite behavior was observed for the YjhI-based biosensor, as the Hill number evaluated for KDX was in the range of 2.7, whereas that of OHB was ≈ 1.25 (Table 1). These results suggested that although both effectors may interact with the same protein, they differentially modulate cooperative interactions within these proteins. The dynamic range (DR), expressed as the fold induction of fluorescence relative to baseline, for the natural (KDX) and artificial (OHB) effectors could be also extrapolated from the dose–response curves. They revealed that the dynamic range of the YjhI-based biosensor for OHB and KDX was about 2 times higher than that of XynR-based biosensor (i.e., in the range of 50-fold versus 25; Table 1).

**Table 1:** Characteristic performance of XynR and YjhI transcription factors towards their metabolic effectors

Effector	XynR*			YjhI*		
	n^H^	K_0.5_	DR	n^H^	K_0.5_	DR
OHB	2.05 ± 0.7	0.27 ± 0.05	25 ± 5.0	1.25 ± 0.25	0.36 ± 0.07	48 ± 6.0
KDX	1.13 ± 0.3	1.0 ± 0.04	25 ± 5.0	2.7 ± 0.8	0.32 ± 0.04	52 ± 8.0

*Values are the mean ± SD of four biological replicates. n^H^ means Hill number, *DR* means dynamic range

### Development of a OHB biosensor

**Fig. 8:**
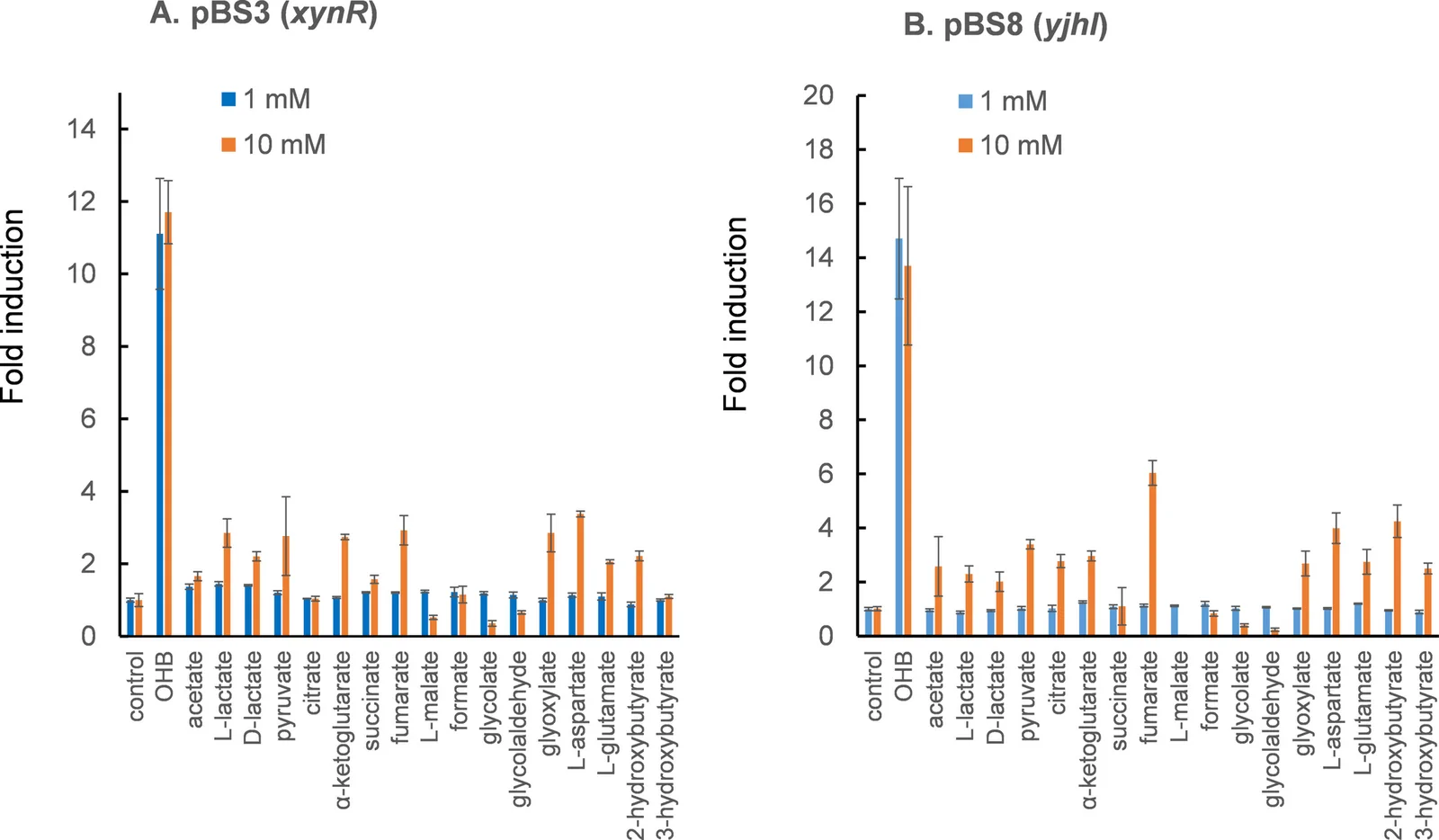
The XynR-based biosensor is specific to OHB. **A** is reported the fluorescence measured after 12-h incubation of the strain MGΔ7 transformed with pBS3 in the presence of various organic and amino acids added at 10 mM except for OHB (1 mM). **B** is the same as **A** except that different sugar acids were added at 20 mM. Dashed line represented the induction fold of control which is 1.0. Data are the mean ± SD (shown by bar on the histogram) of three biological replicates

From the results presented above, it turned out that both transcription factors XynR and YjhI could be exploited for the design of an OHB-responsive biosensor. However, to ensure the practical use of these biosensors, we had to verify the specificity to this unnatural metabolite with respect to other metabolites present in bacterial cells, particularly organic and amino acids. These metabolites were tested at a low (1 mM) and high concentration (10 mM), and the fluorescence signal that can be triggered by these compounds was compared to that obtained upon addition of OHB at 1 mM. As shown in Fig. 8, all the metabolites tested at 1 mM had no effect on both XynR and YjhI-based biosensor. When assayed at 10 mM (Figure S5 reports change in fluorescence over time), metabolites that have like OHB, a ketone function at C2, such as pyruvate, glyoxylate, α-ketoglutarate, and aspartate caused a two- to fourfold induction of fluorescence, with both XynR and YjhI-based biosensors. A two- to fourfold induction was also recorded with organic acids having an α hydroxyl function, such as D and L-lactate or 2-hydroxybutyrate. Less obvious was the finding of a three- to sixfold induction caused by 10 mM fumarate. On the other hand, the basal fluorescence of these two biosensors was lower than the control in the presence of high concentration of L-malate, glycolate, and glycolaldehyde, which also harbor an α-hydroxyl group. How these molecules caused such a reduction of the fluorescence is so far unclear but nevertheless, these effects can be considered negligible overall, as they occurred at concentrations well above those experienced in cells (Bennett et al. 2009). We also investigated the effects of sugar acids homologous to D-xylonate as they can be used as carbon substrate. Results of this experiment showed that both XynR- and YjhI-based biosensor were responsive neither to the enantiomer (L-xylonate) nor the stereoisomer (L-arabinonate) of D-xylonate (Figure S6 in suppl Data). The lack of effect of these sugar acids is consistent with the fact that these compounds cannot be metabolized by *E. coli* (Ren et al. 2022). Conversely, the threefold increase in response to D-gluconate could be explained either by its dehydration into 2-keto-3-deoxy-D-gluconate catalyzed by YagF/YagG dehydratase since the fluorescence signal was abolished in a mutant lacking these dehydratases (data not shown). D-Glucuronate and D-threonate were inefficient as the former one can only be metabolized via the Entner-Doudoroff providing D-glucuronate isomerase encoded by *uxaC* (Portalier et al. 1980) is functional, which needs the presence of this sugar acid to be expressed (Mandrand-Berthelot et al. 2004), whereas only L-threonate is reported to be metabolized by *E. coli* (Frazão et al. 2023).

Beside specificity, other relevant properties of a metabolite responsive transcription factor exploited as a biosensor should be to exhibit a wide range of effector concentrations and a large dynamic response characterized by high fold induction of fluorescence (response) relative to the background. These properties can be tuned at first glance through promoter engineering of either the transcription factor and/or the reporter gene *syfp2*. Starting from pBS3 as the reference plasmid bearing the XynR-based biosensor, we generated a series of plasmids in which either the native RBS in the XynR-promoter was replaced by a synthetically optimized RBS_01_ according to RBS calculator (Salis 2011) or the native promoter of XynR was replaced with iGEM promoters of decreasing strength, while retaining the same RBS_01_ in these synthetic promoters. These different constructs did not result in a better dynamic range to OHB as compared to the original construct in pBS3 (see Figure S8 in suppl. data). It is clear that other modifications, such as engineering the transcription factor XynR to broaden the detection range or sensitivity to OHB, should be considered, but this work is beyond the scope of this study.

### Use of the XynR- and YjhI-based biosensor to screen for OHB producing enzymes

**Fig. 9:**
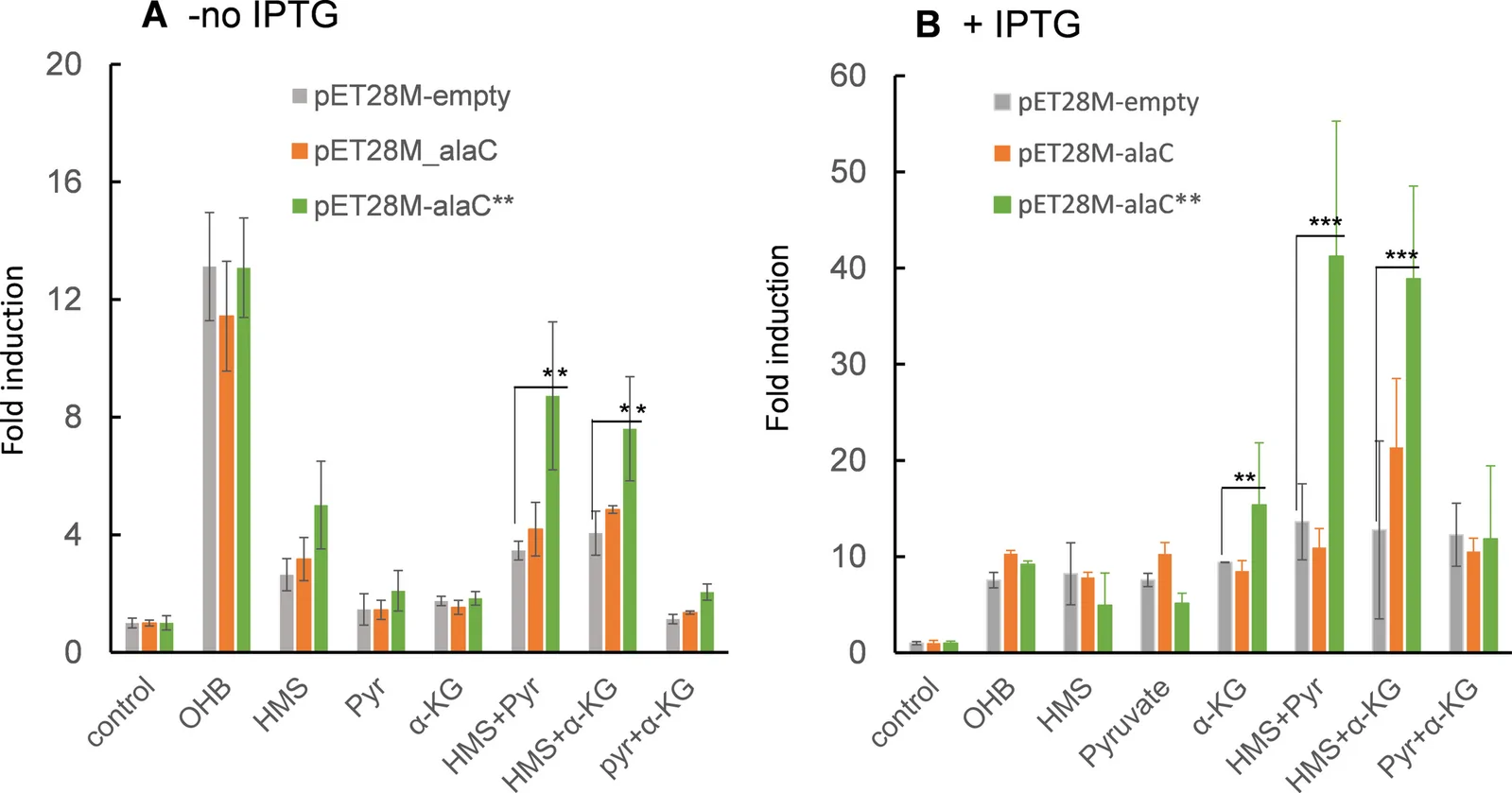
Use of the YjhI-based biosensor to screen for transaminase in OHB formation. The strain MGΔT (Table 1) was co-transformed with pBS8 and with pET28M-empty (grey histograms), pET28M expressing wild type *AlaC* (pET28M_alaC; orange histograms) or a variant AlaC^A142P Y245D^ (pET28M_alaC**; green histograms). After pre-culture in LB with appropriate antibiotic, either no IPTG (panel **A**) or IPTG (panel **B**) was added to the culture when reached OD_600_ 0.6 and incubated for another 4 h before collecting and resuspension in PBS buffer as described in the “Material and methods” section. After addition of the various compounds at 5 mM, except OHB, which was used as the positive control and added at 1 mM, the fluorescence was monitored for 16 h. Data reported are the fold induction determined after 12 h of incubation at 37 °C and values reported are the mean ± SD (shown by bars on the histogram) of four biological replicates. Abbreviation: *OHB* 2-oxo-4-hydroxybutyrate, *Pyr* pyruvate, *HMS* L-homoserine, *α-KG* alpha ketoglutarate

The compound OHB is the most proximal intermediate in the production of 2,4-DHB, a chemical platform for the production of several added-value products (Francois 2023). The synthesis of OHB can be obtained by transamination of L-homoserine with pyruvate or α-ketoglutarate as the co-substrate (Bouzon et al. 2017; Walther et al. 2018). However, the catalytic efficiency of this enzyme is more than 100 times lower than that of other enzymes in these pathways. Improving the activity of these enzymes is therefore mandatory in order to increase the flux in the pathway and consequently increase the rate of 2,4-DHB production. In that frame, a biosensor able to monitor the formation of the product of the reaction catalyzed by the enzyme, such as OHB in our case, could be an appropriate tool for high throughput screening of more active transaminases. We thus decided to validate the use of the XynR/YjhI-based biosensor to screen for transaminase activity using the variant of AlaC^A142P Y245D^ as a positive control since this variant turned out to be the most L-homoserine transaminase to produce OHB, albeit at a still low catalytic efficiency of ≈ 500 M^−1^ s^−1^ ((Bouzon et al. 2017), our unpublished data). To validate the screen, we used the strain MGΔT which was deleted for *tyrB*, *ilvE, ybdL*, and *alaC* to get rid of several alternate transaminases exhibiting even a weak activity on L-homoserine (Walther et al. 2018, 2017). We tested the YjhI-biosensor through co-transformation of this strain with pBS9 and either pET28M (empty), used as control, pET28M carrying *alaC* (coding for wild-type AlaC), and pET28M carrying alaC** (coding for the variant AlaC^A142P Y245D^). Since these genes were under the control of an IPTG-inducible promoter, part of the culture was treated for 4 h with 0.5 mM IPTG before addition of the various metabolites. As reported in Fig. 9, the fold induction of fluorescence of IPTG-untreated cultures in response to homoserine, pyruvate or α-ketoglutarate was in the range of 1.5 to 4, and always lower than in response to 1 mM OHB use as control. However, a statistically significant induction fold of 8 relative to the control was measured in the strain carrying pET28M-alaC**, indicating that even in the absence of IPTG, the genes in these plasmids were expressed and moreover that AlaC^A142P Y250D^ variant was more active than AlaC to produce OHB. Also, the weak but significant increase in fluorescence in strain expressing the empty plasmid pET28M in response to homoserine alone or with pyruvate or α-ketoglutarate could be ascribed to some endogenous transaminases such as the one encoded by *aspC* which has been reported to have a weak OHB producing activity (Walther et al. 2018). More importantly, the treatment of the culture with IPTG prior addition of the various metabolites clearly validated the biosensor tool since a 40-fold induction of fluorescence was recorded after addition of L-homoserine with either pyruvate or α-ketoglutarate in strain expressing the AlaC^A142P Y250D^ variant, which is close to the maximal dynamic response of OHB (see Fig. 7). However, a significant eightfold induction had already been measured in the strain expressing the empty vector (pET28M) when incubated with homoserine, pyruvate, or α-ketoglutarate alone; this could pose challenges for the screening process and therefore requires, first and foremost, optimization of the biosensor to reduce this background noise. Notice that we also evaluated the XynR-based biosensor but it turned out that the results obtained were inconsistent, with an apparent interference of IPTG on the expression of pBS3 since the fluorescence response to OHB was strongly impaired in cells treated with IPTG (data not shown).

**Fig. 10:**
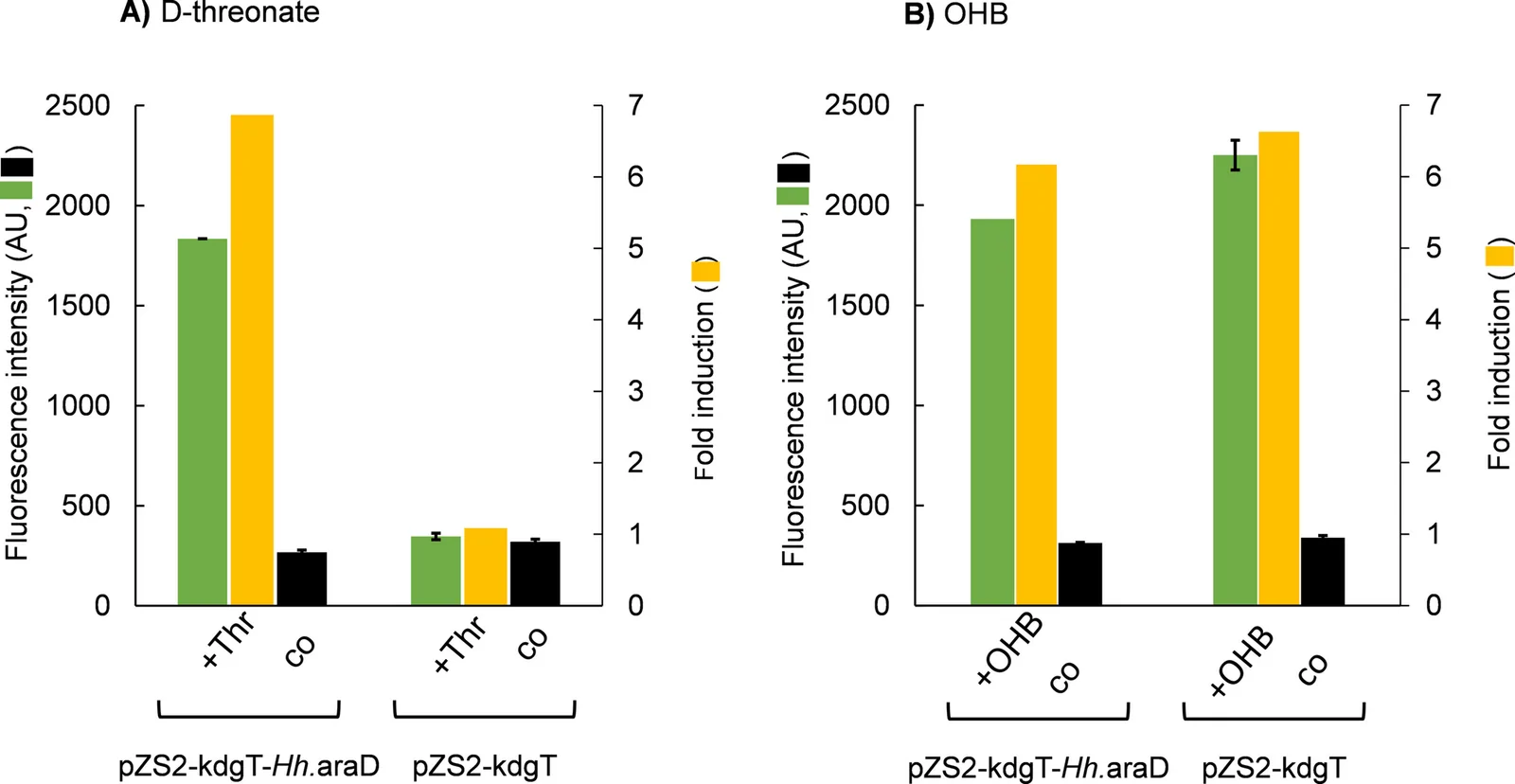
Use of the XynR-based biosensor to screen for threonate dehydratase catalysing the formation of OHB. The strain MGΔ*7* was co-transformed with pBS3 and pZS2-kdgT or pZS2-kdgT *Hh*araD which encodes the threonate dehydratase from *Herbaspirillum huttiense*. Induction of these genes and incubation of the cells with D-threonate (10 mM) are described in the “Material and methods” section. The fluorescence was determined by flow cytometry after 12-h incubation. Panel **A** shows the experiment in which D-threonate (10 mM) was added, and panel **B** with OHB (1 mM). Data are the mean ± SD (shown by bar of the histogram) of two biological replicates, each containing 3 technical replicates

We then tested the D-threonate dehydratase activity which was derived from the promiscuous D-arabinonate dehydratase of *Herbaspirillum huttiense* (*HharaD*) (Watanabe et al. 2019). This enzyme was shown to synthetize OHB by dehydration of D-threonate in a conceived synthetic pathway starting from ethylene glycol and ending with the formation of 2,4-DHB (Frazão et al. 2023). For this validation, MG1655 strain was co-transformed with pBS3 (XynR-based biosensor) and either pZS2 carrying *HharaD* with *kdgT* encoding a transporter of *Cupriavidus necator* reported to facilitate the import of D-threonate, or with pZS2 carrying only *kgdT*. Remarkably, the addition of D-threonate to bacterial cells expressing *HharaD* encoding this threonate dehydratase resulted in a significant increase in fluorescence signal that was equivalent to that obtained after the addition of 1 mM OHB, whereas the fluorescence of cells lacking this gene remained at a basal level after the addition of the sugar acid (Fig. 10). Of note, the assay was not carried out with the YjhI-based biosensor due to the scarcity of D-threonate.

## Discussion

The construction of biosensors dependent on the transcription factors XynR and YjhI enabled us to resolve two major problems concerning the induction of the D-xylonate catabolic pathway in *E. coli*. First, the natural effector of these two transcription factors that induce the catabolic genes present on the *yag* and *yjh* operons is not D-xylonate, but the intermediate KDX, which is formed by the dehydration of D-xylonate by D-xylonate dehydratase encoded by *yagF* and *yjhG*. Moreover, our results suggested that in vivo YagF is more active than YjhG. This higher activity could be explained either by a higher expression of *yagF* than *yjhG*, or by a higher affinity of YagF for D-xylonate. To date, only kinetic data have been reported for the D-xylonate dehydratase YjhG, showing a K_M_ of 4.88 mM and a relative weak k_cat_/K_M_ of 66 M^−1^ s^−1^ (Jiang et al. 2015b). Also, our work reported no cross-talk between transcription factor to regulates the two operons encoding D-xylonate metabolic genes, which agreed with the fact that the location of the binding of these two transcription factors on the promoter is clearly distinct.

The other part of the work was to examine whether XynR and/or YjhI are also responsive to the non-natural metabolites 2,4-DHB and/or OHB to repurpose them as biosensor tools to improve the synthetic pathways of the platform molecule 2,4-DHB (Francois 2023). Our data showed that OHB can effectively play the same role as KDX on these two transcription factors, most likely because OHB and KDX have very similar structure but chemically different by the number of carbon chain. Based on data with other organic and amino acids used to test the specificity of Xynr and YjhI to OHB and KDX, it can be proposed that the minimal structure recognized by these TFs required the functional carboxyl group, a keto function on the carbon α and a hydroxy group on the carbon γ. Furthermore, the 3D structures of XynR and YjhI are very similar, as predicted by AlphaFold-2, which supports the idea that they may respond to these two effectors. Despite this structural similarity, the kinetic parameters—namely the response threshold (K_0.5_) and the Hill coefficient (n^H^)—were very different between these two TFs for KDX and OHB, as well as between KDX and OHB for each transcription factor. Thus, the response threshold for OHB in XynR was 3 to 4 times lower than that measured for KDX, whereas the opposite was determined for YjhI. Furthermore, a Hill coefficient of ≈ 2.05 obtained with OHB suggests that the action of this effector to release the repression of genes caused by XynR may occur through a positive cooperativity, requiring a probable dimerization of XynR. Similarly, the binding of YjhI to the promoter very likely implied a positive cooperativity involving at least a dimerization of this protein under the action of KDX (e.g., n^H^ ≈ 2.7). In conclusion, each transcription factor exhibits specific properties regarding its interaction with the effectors KDX and OHB and its promoter binding, which necessitates an in-depth study of their structure–function relationship that goes beyond the scope of this work.

In previous works, we developed 4 different synthetic pathways leading to the production of 2,4-DHB from C1 to C6-renewable carbon sources (Frazão et al. 2023; Walther et al. 2017, 2018). This synthon can be considered a unique bio-based platform molecule capable of giving rise to several value-added molecules, including a hydroxylated derivative of methionine, 1,3-propanediol, 3-hydroxypropionic acid, 1,2,4-butanetriol, and biopolymers (Francois 2023; Pascouau et al. 2023a, 2023b). Two of these four pathways, which have proven to be the most suitable for industrial application, rely on the formation of the intermediate OHB by promiscuous enzymes whose catalytic efficiency is far too low, significantly hampering the productivity and yield of 2,4-DHB production. An OHB-sensitive biosensor could be an appropriate method, which combined with flow cytometry coupled with cell sorting (FACS), would enable the selection of enzyme variants with higher catalytic activity. The XynR-based biosensor can be optimally designed for use in this context, as it features a repressed-repressor architecture that enables positive selection, i.e., the signal response is triggered only when the target metabolite is produced. Our data aligned in part with these requirements. We validated the potential value of this OHB-sensitive biosensor as a screening tool for threonate dehydratase, the rate limiting enzyme in the synthetic pathway yielding to DHB from ethylene glycol as the carbon source (Frazão et al. 2023). However, it was not possible to use this XynR-based biosensor as a screening method for L-homoserine transaminase, which catalyzes the rate-limiting step in the formation of DHB from glucose via the aspartate-homoserine pathway (Walther et al. 2018), whereas the YjhI-based biosensor, which has an activator-activated architecture, turned to be a potential tool, albeit requiring optimization to reduce background fluorescence. Finally, our OHB-sensitive biosensors differ significantly from the “HOB biosensor” recently described by Schann et al. (2024), as the latter is in fact a formaldehyde growth-based sensor, which enables growth on this C1 carbon of a strain deficient in the natural aspartate-homoserine pathway through the expression of promiscuous aldolases that condense it with pyruvate to form OHB, which is transaminated to L-homoserine via promiscuous transaminases.

## Supplementary Information

Below is the link to the electronic supplementary material.

ESM 1 PDF (961 KB)

## Data Availability

No datasets were generated or analysed during the current study.
