# Supplementary material for: 2-Keto-3-deoxy-D-xylonate and 2-oxo-4-hydroxybutyrate as natural and artificial effectors of transcription factors regulating D-xylonate operons in E. coli
Source: Appl Microbiol Biotechnol. 2026 May 1;110:184. doi: 10.1007/s00253-026-13840-y (PMC13279387; doi:10.1007/s00253-026-13840-y)
Supplement: Supplementary file 1 [file 253_2026_13840_MOESM1_ESM.pdf]

Supplementary data

2-keto-3-deoxy-D-xylonate and 2-oxo-4-hydroxybutyrate as a natural and artificial effector of the transcription factors XynR and YjhI

Thibault MALFOY[1*], Ceren ALKIM[1*], Julie FREDONNET[2], Juan LAJARIN-HERNANDEZ[1] and Jean Marie FRANCOIS[1,2$]

[1]Toulouse Biotechnology Institute, UMR INSA -CNRS5504 and UMR INSA-INRAE 792, 135 avenue de Rangueil, 31077 Toulouse, France

[2]Toulouse White Biotechnology, UMS INRAE-INSA-CNRS, 135 Avenue de Rangueil, 31077 Toulouse, France

Email address

Thibault Malfoy	malfoy@insa-toulouse.fr
Ceren Alkim	alkim@insa-toulouse.fr
Juan Lajarin-Hernandez	lajarin-hern@insa-toulouse.fr
Julie Fredonnet	Julie.fredonnet@inrae.fr
[$]Jean M François	fran_jm@insa-toulouse.fr or jean-marie.francois@inrae.fr

Table S1: Strains used in this study

Strain name	Description -genotype	source
MG1655	F- lambda- *ilvG- rfb-50 rph-1*	ATCC n°47046
NEB®5α	*fhuA2 Δ(argF-lacZ)U169 phoA glnV44 Φ80 Δ(lacZ)M15 gyrA96 recA1 relA1 endA1 thi-1 hsdR17*	NEB competent *E. coli*
BL21 (DE3)	*fhuA2 [lon] ompT gal (λ DE3) [dcm] ΔhsdS*<br>*λ DE3 = λ sBamHIo ΔEcoRI-B int::(lacI::PlacUV5::T7 gene1) i21 Δnin5*	NEB
BW25113	*Δ(araD-araB)567 ΔlacZ4787(::rrnB-3) λ- rph-1 Δ(rhaD-rhaB)568 hsdR514*	CGSC#7636 (Baba et al. 2006)
MGΔ7	MG1655 *ΔyagE::FRT ΔyjhH::FRT Δeda::FRT ΔdgoA::FRT ΔyfaU::FRT ΔgarL::FRT ΔmhpE*	This study
MGΔLO	MG1655 *Δdld::FRT ΔykgEFG::FRT ΔlldD::FRT*	This study
MGΔT	MG1655 *ΔybdL ΔtyrB ΔilvE ΔalaC*	This study
MGΔ4	MG1655 *ΔlysC ΔthrA Δasd ΔmetL*	This study
MGΔF	MG1655 *ΔyagF ::FRT*	This study
MGΔG	MG1655 *ΔyjhG ::FRT*	This study
MGΔFG	MG1655 *ΔyagF ::FRT ΔyjhG ::FRT*	This study
MGΔX	MG1655 *ΔxynR ::FRT*	This study
MGΔJ	MG1655 *ΔyjhI ::FRT*	This study
MGΔXJ	MG1655 *ΔxynR ΔyjhI*	This study
MGΔ7 ΔXJ	MG1655 *ΔxynR ::FRT ΔyjhI ::FRT ΔyagE::FRT ΔyjhH::FRT Δeda::FRT ΔdgoA::FRT ΔyfaU::FRT ΔgarL::FRT ΔmhpE*	This study
MGΔTΔA	MG1655 *ΔybdL ΔtyrB ΔilvE ΔalaC Δasd*	This study

Table S2: Plasmids constructed and used in this study

Plasmid	Short-Description	source
pET28a		Novagen
pZA33	Clm^R, p15A ori, promoter P_{A1} lacO1	Expressys
pREP22	pZE13 derivative, Amp^R; ColE1 ori, promoter P_{yqhD} hybrid: $RBS_{O1:}$:*syfp2*	(Frazao et al. 2018)
pCP20	Amp^R, Clm^R, pSC101 ori	(Cherepanov and Wackernagel 1995)
pBS0	pZA33 derivative, Clm^R, p15A ori, promoter P_{yagE} : RBS_{O1}::*syfp2*	This study
pBS2	pZE13 derivative, Amp^R; ColE1 ori, promoter P_{yagE} : RBS_{O1}::*syfp2*, promoter P_{xynr}:RBS_{xynr}: *xynR*	This study
pBS3	pZA33 derivative, Clm^R, p15A ori, promoter P_{yagE} : RBS_{O1}::*syfp2*, promoter P_{xynr}:RBS_{xynr}: *xynR*	This study
pBS5	pZE13 derivative, Amp^R; ColE1 ori, promoter P_{yagE} : RBS_{O1}::*syfp2*, promoter P_{xynr}:RBS_{xynr}: *xynR*; promoter BBa_J23106:*lldD*	This study
pBS7	pZA33 derivative, Clm^R, p15A ori, promoter P_{yjhI} : RBS_{O1}::s*yfp2*	This study
pBS8	pZA33 derivative, Clm^R, p15A ori, promoter P_{yjhI} : RBS_{O1}::*syfp2*, promoter P_{yjhI}:RBS_{yjhI}: *yjhI*	This study
pBS6	pZA33 derivative, Clm^R, p15A ori, promoter P_{yagE} : RBS_{O1}::*syfp2*, promoter P_{yjhI}:RBS_{yjhI}: *yjhI*	This study
pBS9	pZA33 derivative, Clm^R, p15A ori, promoter P_{yjhI} : RBS_{O1}::*syfp2*, promoter P_{xynr}:RBS_{xynR}: *xynR*	This study
pBS10	pZE13 derivative, Amp^R; ColE1 ori, promoter P_{yagE} : RBS_{O1}::*syfp2*, promoter P_{xynr}:RBS_{xynr}: *xynR*; promoter BBa_J23106:*yagE*	This study
pET28M_empty	pET28a with T5 promoter_empty	This study
pET28M_alaC	pET28a with T5 promoter carrying wild type *alaC*	This study
pET28M_alaC**	pET28a with T5 promoter carrying mutated *alaC* expressing $AlaC^{A142P\ Y275D}$ variant	This study

Table S3: Primers used in this study

Oligonucleotide	Sequence
TM4	tcgggctttgTCAGGCCGGGTAGCCCAG
TM5	cccggcctgaCAAAGCCCGAAAGGAAGC
TM7	ctagctcgaGGACTGCACCGAGCCATC
TM8	tgcaagcttGAGATCTCCTTGCTGAATCATTTTG
TM9	catgagcggatacatatttg
TM10	aactaagatcggtactacgc
TM11	ggataacgcaggaaagaacaAAACCGGATGCATACGATTAAGGCG
TM12	TGTGAGCAAAAGGCCAGC
TM13	TGTTCTTTCCTGCGTTATCC
TM14	ttgctggccttttgctcacaGCGCCGCTACAGGGCGCG
TM15	ctatggaaaaacgccagc
TM16	gttgtttgtcggtgaacg
TM63	tttacggctagctcagtcctaggtatagtgctagcACTAGTgaaagaggagaaatactag
TM64	ctagtatttctcctctttcACTAGTgctagcactatacctaggactgagctagccgtaaa
TM65	ggggttccgcgcacatttccccgaaTTTACGGCTAGCTCAGTC
TM66	ggaaataatcatCTAGTATTTCTCCTCTTTCAC
TM67	ggagaaatactagATGATTATTTCCGCAGCC
TM68	tcgtgaggatgcgCTATGCCGCATTCCCTTTC
TM69	aatgcggcatagCGCATCCTCACGATAATATC
TM70	gtttcttagacgtcaggtggcacttGTTTCTACTGGTATTGGC
TM71	AAGTGCCACCTGACGTCTAAG
TM72	TTCGGGGAAATGTGCGCG
TM146	tggccttttgctc
TM233	CCTATATCGCCGACATCAC
TM234	GGATCCGAATTCGAGCT
TM242	aggcgccagcTAAAAATAGGCGTATCACG
TM243	attcggatccCATAGTTAATTTCTCCTCTTTAATG
TM250	cttcttaaagttaaaCgctagcataata
TM251	gagatataccATGAAAGCCAAC
TM252	TTTATGGCTAGCTCAGTCC
TM253	AAGACGAAAGGGCCTC
JFR64	GTGAGCAAAAGGCCAGACCTAGGGATA TATTCCGCTTCC
JFR65	CGTCAGGTGGCACTTttagacgtcgga attgccag
JFR66	CAGATTTCGTGATGCTTGTC
JFR67	ATGGTGAAAGTTGGAACCTC
917	TATTAATTAGAGCTCATGGCTGACACTCGCCCTGAACGTCGCTTTA
926	TATTAATTAAGCTTTTATTCCGCGTTTTCGTGAATATGTTTGCTGCTGGCG
seq_R	GTTCTTTCCTGCGTTATCCcc
seq-F	atgCCGATTATTCAGTCTGTTGAAC
seq_ xynR rbs01_R	CTGAATAATCGGcatTCCAAACCTCCTCGAAAACGT
seq_xynR rbs01_J23119_R	CTGAATAATCGGcatTCCAAACCTCCTGCTAGCATTATAC
seq-xynR rbs01_J23100_R	CTGAATAATCGGcatTCCAAACCTCCTGCTAGCAC
seq_xynR rbs01 J23106_R	CTGAATAATCGGcatAATGCTGGCATGTCCACG
seq_xynR rbs01_J23104_R	CTGAATAATCGGcatTCCAAACCTCCTGCTAGCAC

seq-xynR_F	AACGCAGGAAAGAACAAAACCGGATGCATACGATTAAGG
1781-gRNA - lacZ	ACTAGTATTATACCTAGGACTGAG
2996-oligo-pTarget-asd	GTCCTAGGTATAATACTAGTATCGGCTGGCGCGGTATGGTGTTTTAGAGCTAGAAATAGC
2999-asd-vrf-F	ATCTGATTCGGGTACTGATG
3000-asd-vrf-rev	GATAATAGCCAGGCATCCAT
3116-vrf-yjhI-F	CCTCATTCAATAAAGTGATAAGT
3117-vrf-yjhI-R	CGATCTGCTGGTAATAGT
3131-amp-syfp2-F	GGACTGCACCGAGCCATCTT
3132-amp-syfp2-R	CGGCGGATTTGTCCTACTCA
3133-remove-2genes-F	CTGAAAGGAGGAACTATATCCGGATTGG
3134-remove-2genes-R	TCGAggtgaagacgaaagggc
3135-inf-pBS0-F	tcgtcttcaccTCGAGACTGCACCGAG
3136-inf-pBS0-R	AGTTCCTCCTTTCAGcggcggatttgtcctact
3126-xynR-remove-F	CTGAAAGGAGGAACTATATCCGGATTGG
3127-xynR-remove-R	GTTCTTTCCTGCGTTATCCcct
3128-yjhI-infusion-F	AACGCAGGAAGAATCCACACACTACGATGTTGAACA
3129-yjhI-infusion-R	AGTTCCTCCTTTCAGCAAAAAACCCCTCAAGACCCGT
3130-amp-yjhI-F	ATCCACACACTACGATGTTG
3137-ter7-pour yjhI-R	CAAAAAACCCC………CCTTTTCGTAAGTGC*
3146-enlever-yagE-F	AAGCTTTCCAACTTAAATACAAGGAAAATAAGGAG
3147_pyjhI_inf_F	TCGTCTTCACCTCGATCCACACACTACGATGTTGCAACA
3149_pBS8_inf_R	TAAGTTGGAAAGCTTAATGGCTCCTCCTTGCTCATG
3150_yagF_vrf_F	CGCAGATGTATCAGCTGGAT
3151_yagF_vrf_R	TGCCGAATTACAACATCGTG
3152_yjhG_vrf_F	ATACAGCATGCAGTGTGTCG
3153_yjhG_vrf_R	GATGGCGACAGCAATCGAGA
3154_pBS7_over_F	TATCCACAGAATCAGGGGATAACGCAGGAAAGAACCTGAAAGGAGGAACTATATCCGGATTGGCG
3155_pBS7_over_R	GTTCTTTCCTGCGTTATTCCCCTGATTCTGTGGATAACCGTATTACCGCCT
3159_del_v_xynR_F	GTATCAGGTGAACGCGCAGA
3160_del_v_xynR_R	GGGCTGACCTGCTACTACAA
3164_del_v_yjhI_F	CTGTAGCAACACTATCATGT
3165_del_v_yjhI_R	GATCTGCTGGTAATAGTCGT
3168-remov-lldD-F	CGCATCCTCACGATAATATCcgggta
3173-inf-pBS10-F	GAGGAGAAATACTAGatgCCGCAGTCCGC
3174-inf-pBS10-R	TATCGTGAGGATGCGtcaGCAAAGCTTGAGCTGTTGCAG
3175-rem-lldD-p10-R	CTAGTATTTCTCCTCTTTCACTAGT

Table S4: Genetic modifications of the promoter and RBS of the XynR-based biosensor

name		Synthetic DNA fragments
pBioS-JF1 (pBS3)	seq Pxynr:RBS_{xynR}	AAAACCGGATGCATACGATTAAGGCGGTGATGATGGCAACGCTTGGGGAGTGATTGGGTCCGCTGCGCGTTGGTGCCCTCACCCCGGCCCTCTCCCACAGGGAGAGGGAGAACACCGGCTCCATTTCATTGATTTTTCATCCCGAAAAAGGTACGTTTTCGCCTTAATTCCAGCGTGGACATGCCAGCATT
pBioS-JF2	seq PxynR : RBS_{O1}	AAAACCGGATGCATACGATTAAGGCGGTGATGATGGCAACGCTTGGGGAGTGATTGGGTCCGCTGCGCGTTGGTGCCCTCACCCCGGCCCTCTCCCACAGGGAGAGGGAGAACACCGGCTCCATTTCA**ttgatttttcatcccgaaaaaggtacgttttcg**AGGAGGTTTGGA
pBioS-JF3	seq PJ23119: RBS_{O1}	AAAACCGGATGCATACGATTAAGGCGGTGATGATGGCAACGCTTGGGGAGTGATTGGGTCCGCTGCGCGTTGGTGCCCTCACCCCGGCCCTCTCCCACAGGGAGAGGGAGAACACCGGCTCCATTTCA**ttgacagctagctcagtcctaggtataatgctagc**AGGAGGTTTGGA
pBioS-JF4	seq Pj23100:RBS_{O1}	AAAACCGGATGCATACGATTAAGGCGGTGATGATGGCAACGCTTGGGGAGTGATTGGGTCCGCTGCGCGTTGGTGCCCTCACCCCGGCCCTCTCCCACAGGGAGAGGGAGAACACCGGCTCCATTTCA**ttgacggctagctcagtcctaggtacagtgctagc**AGGAGGTTTGGA
pBioS-JF5	seq Pj23104: RBS_{O1}	AAAACCGGATGCATACGATTAAGGCGGTGATGATGGCAACGCTTGGGGAGTGATTGGGTCCGCTGCGCGTTGGTGCCCTCACCCCGGCCCTCTCCCACAGGGAGAGGGAGAACACCGGCTCCATTTCA**ttgacagctagctcagtcctaggtattgtgctagc**AGGAGGTTTGGA
pBioS-JF6	seq Pj23106:RBS_{O1}	AAAACCGGATGCATACGATTAAGGCGGTGATGATGGCAACGCTTGGGGAGTGATTGGGTCCGCTGCGCGTTGGTGCCCTCACCCCGGCCCTCTCCCACAGGGAGAGGGAGAACACCGGCTCCATTTCA**tttacggctagctcagtcctaggtatagtgctagc**AGGAGGTTTGGA

In bold are the sequence of the synthetic promoter. RBS sequence is underlined

Table S5: Expression changes of the D-xylonate metabolic genes from the cryptic CP4-6 prophage regulated by *xynR* (*yagI*) and from the KpLE2 phage-like element regulated by *yjhI* in *E. coli* under fermentation process of 2,4-DHB production or in response to 1 M 2,4-DHB

	**Fermentation condition*** value in fold change			**Toxicity condition**[$] value in fold change	
**Gene name**	T12/T2	T28/T2	T58/T2	T30/T0	T180/T0
xynR(yagI)	0.9	1.08	0.40	1.05	0.65
yagE	11.2	13.6	2.2	2.2	1.3
yagF	8.3	9.1	1.2	2.9	1.8
yagG	1.9	2.2	1.3	2.0	1.9
yagH	1.7	2.1	1.1	2	1.9
yjhI	8.7	7.4	1.3	4.7	1.7
yjhH	3.6	2,0	1,2	1.8	1.1
yjhG	2.5	1,4	1,1	3.1	2.0
yjhF	2,5	1,4	1,1	3,1	2.0

*Fermentation was carried out with a *E. coli* MG1655 strain expressing the homoserine-2,4-DHB pathway (Walther et al. 2018) in a M9 medium with glucose alimentation in a fed batch mode. Samples were taken after 2, 12, 28 and 54 hr of culture for transcriptomic analysis using *E. coli* Agilent gene chips (Alkim et al. 2022). The data are the mean of two technical replicates from 2 biological experiments.

[$]Toxicity experiments were carried out with *E. coli* MG1655 cultivated in M9 medium with 0.4 % glucose. In mid log phase (OD_{600}~1.0), the cells were challenged with 1 M 2,4-DHB ammonium. Samples were taken at 30 and 180 min after addition 1 M 2,4-DHB or 1M ammonium chloride used as control. Transcriptomic analysis was carried out using *E. coli* Agilent gene chips from three biological experiments according to (Alkim et al. 2022).

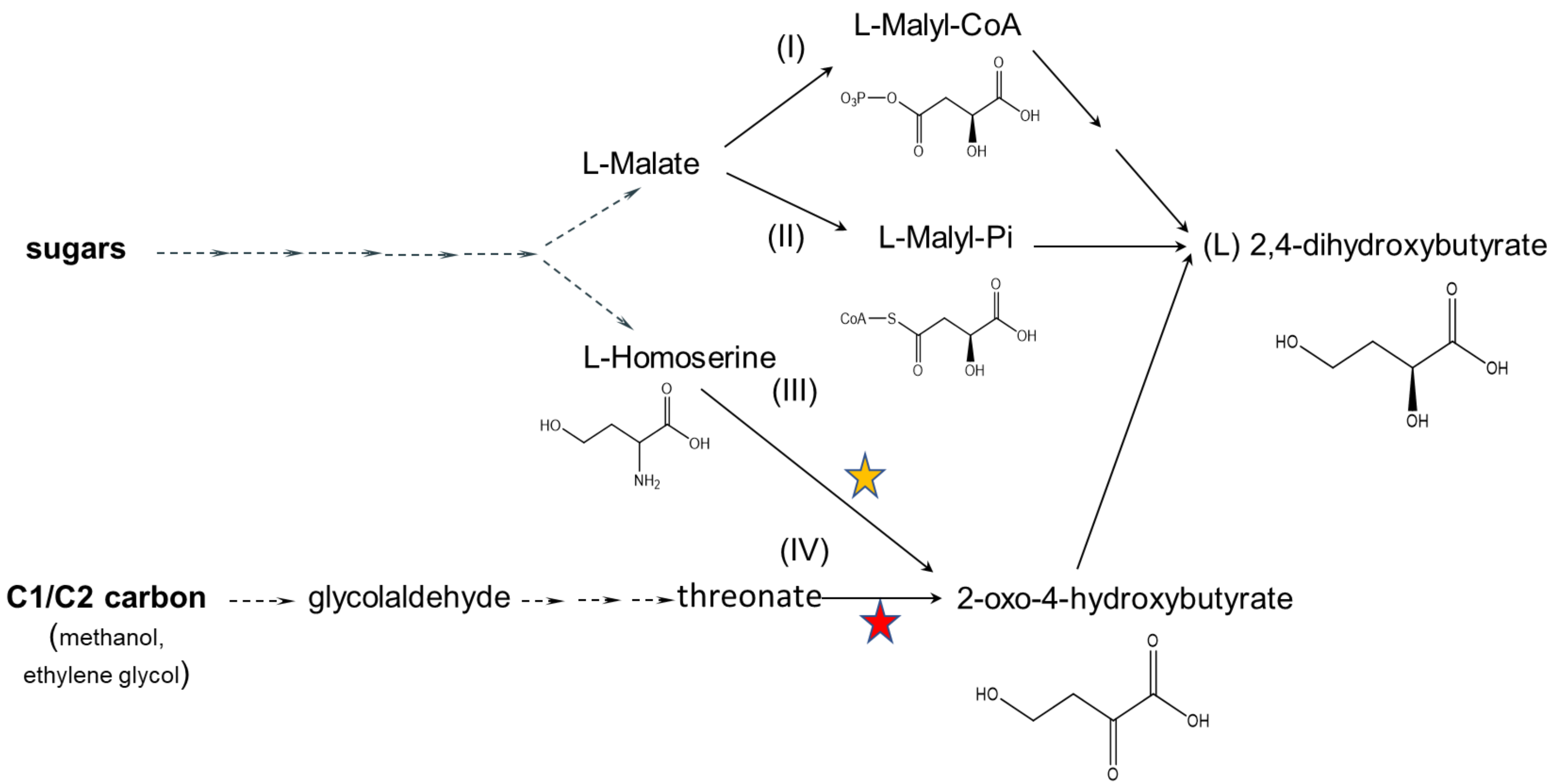

Figure S1: Scheme of the synthetic pathways leading to the production of 2, 4-dihydroxybutyrate from sugars or C1/C2 carbon sources. The yellow and red star indicates transaminase and threonate dehydratase, which are the rate-limiting enzymes for DHB production via the threonate and homoserine pathways, respectively

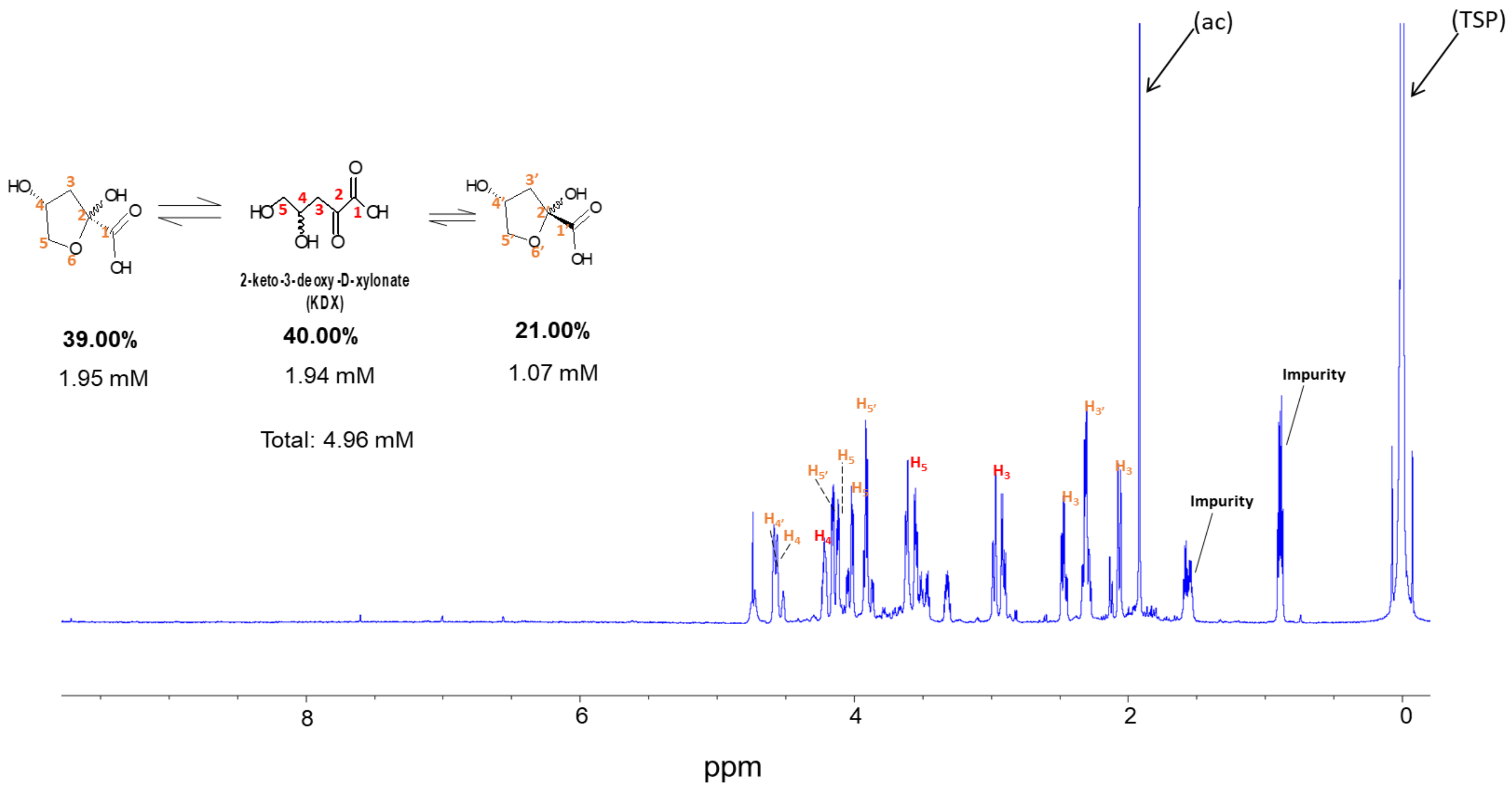

Figure S2: 1^{H}-NMR of 2-keto-3-deoxy D-xylonate. The 1D proton spectrum was acquired on a 800MHz Bruker NEO NMR spectrometer, equiped with a cryogenic QCPI probehead. The sample was prepared in a 3mm NMR tube starting from a commercial 1M KDX solution (2µl) diluted 100-fold in 194µl of D2O and 4µl of Trimethyl silyl propionate (TSP; 500mM) solution in D_2O. The spectrum was recorded with 16k complex points after water pre-saturation with 4 scans and a relaxation delay of 15s. It was Fourier transformed into a matrix of 32k points, after a line broadening with 2Hz. Peaks were integrated manually and compared to the 90mM of TSP protons. The impurity was estimated at 0.8 mM, which gave e purity of KDX in the range of 90%

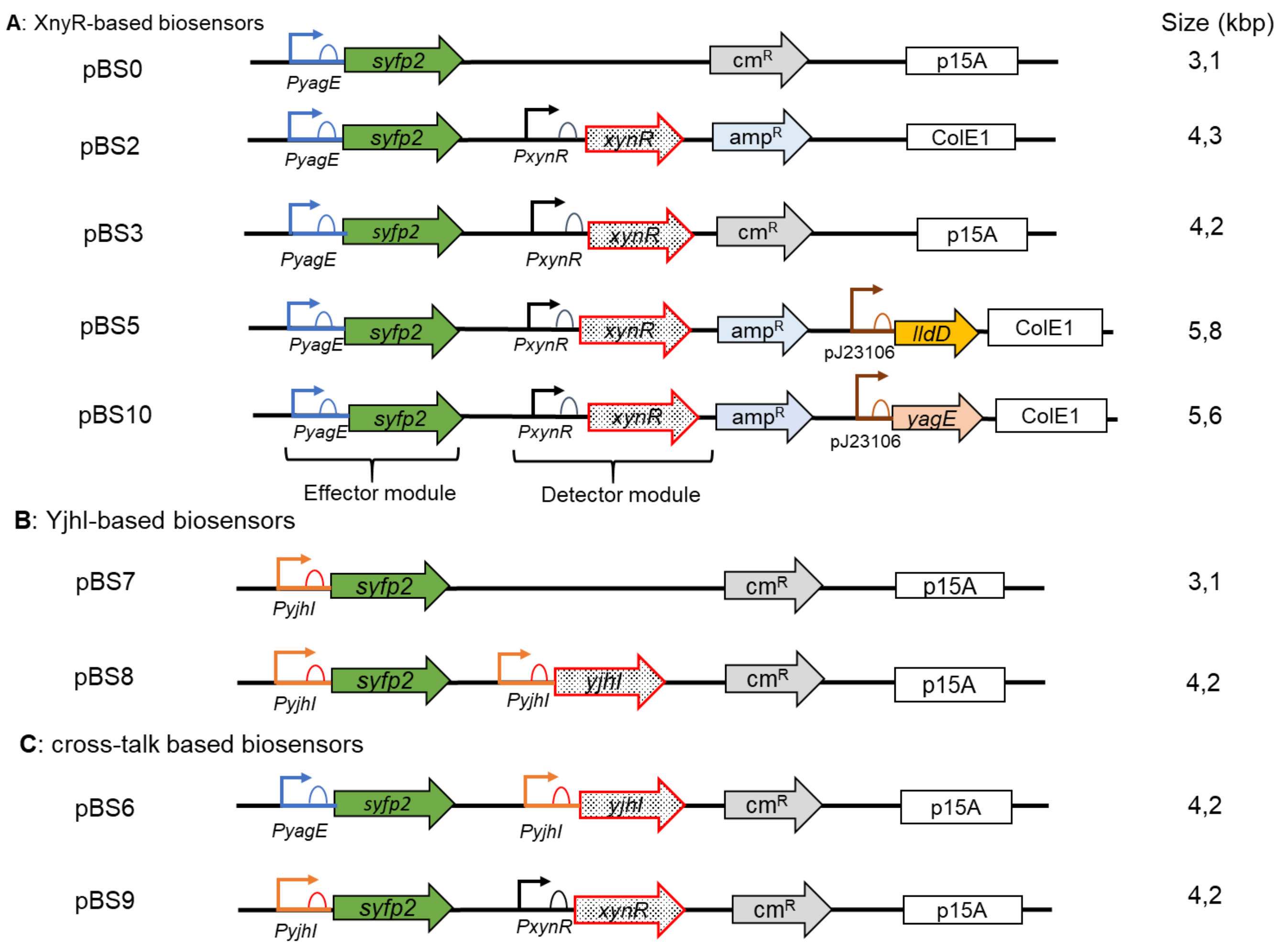

Figure S3: scheme of the transcription factor -based biosensor. (A) Description of the genomic construction of the xynR-based metabolite biosensor carried on medium copy (pBS3) plasmid. The syfP2 reporter gene was flanked upstream by a 300 bp of corresponding to XynR-sensitive promoter region of *yagE*. The gene *lldD* encoding L-lactate oxidase was inserted into pBS2 under the strong synthetic promoter pJ23106 to yield pBS5. The gene *yagE* which encodes a pyruvate-dependent aldolase of the XynR-operon was replaced with the *lldD* gene on pBS5 to yield pBS10. (B) Description of the genomic construction of the YjhI-based metabolite biosensor carried on medium copy (pBS7) plasmid. (C) Description of genomic construction of cross-talk based biosensors.

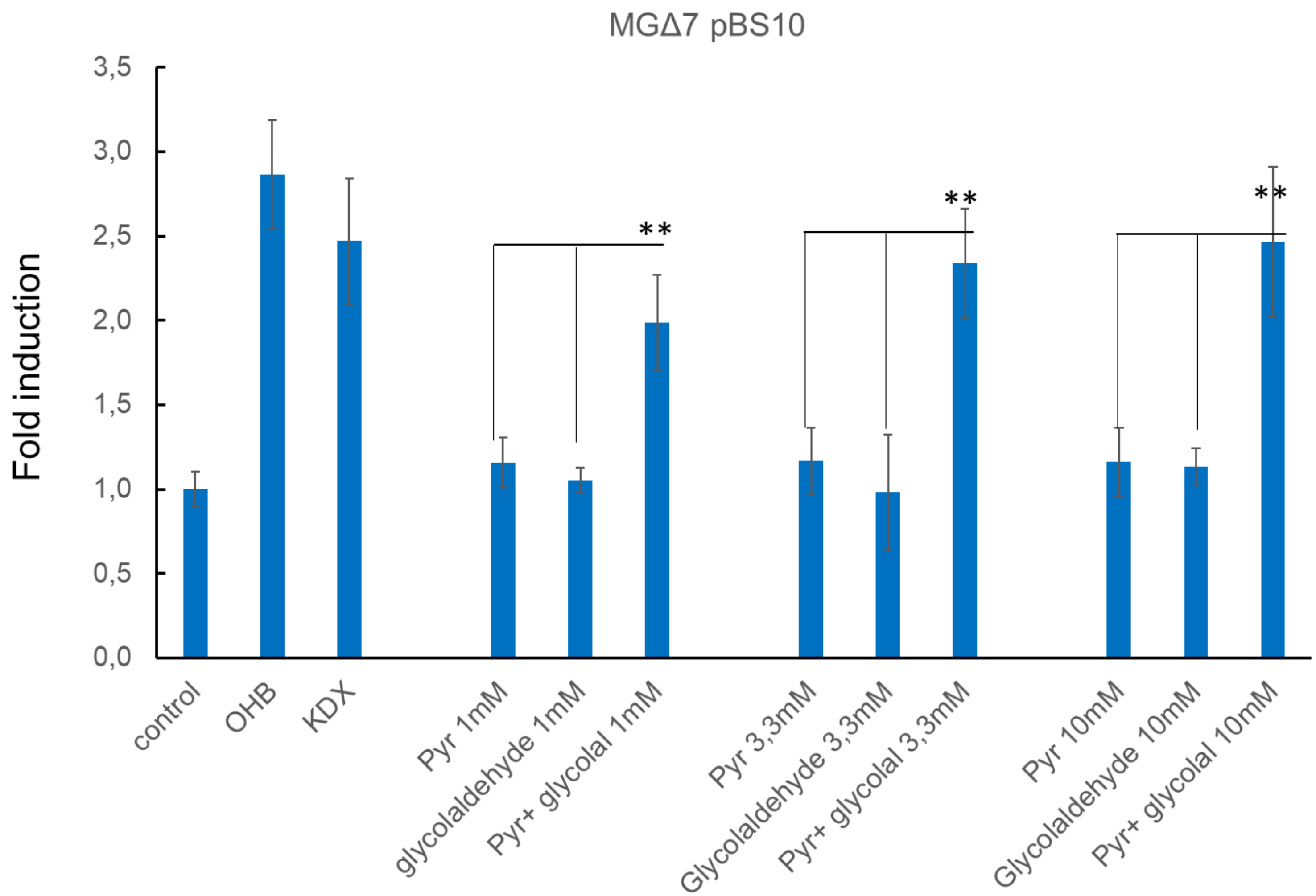

Figure S4: Genetic evidence that KDX is the effector of the XynR transcription factor. Strain MGΔ7 was transformed with the pBS10 vector, which carries the *yagE* gene encoding a pyruvate-dependent aldolase. KDX or OHB at a concentration of 2 mM (used as controls), pyruvate, and glycolaldehyde, either separately or together, at concentrations of 1, 3.3, and 10 mM, were added to the culture of this strain. The fluorescence resulting from *syfp2* expression was measured for 16 hours after the addition of these compounds. The values shown correspond to the induction factor after 12 hours of incubation, calculated from 4 biological replicates.

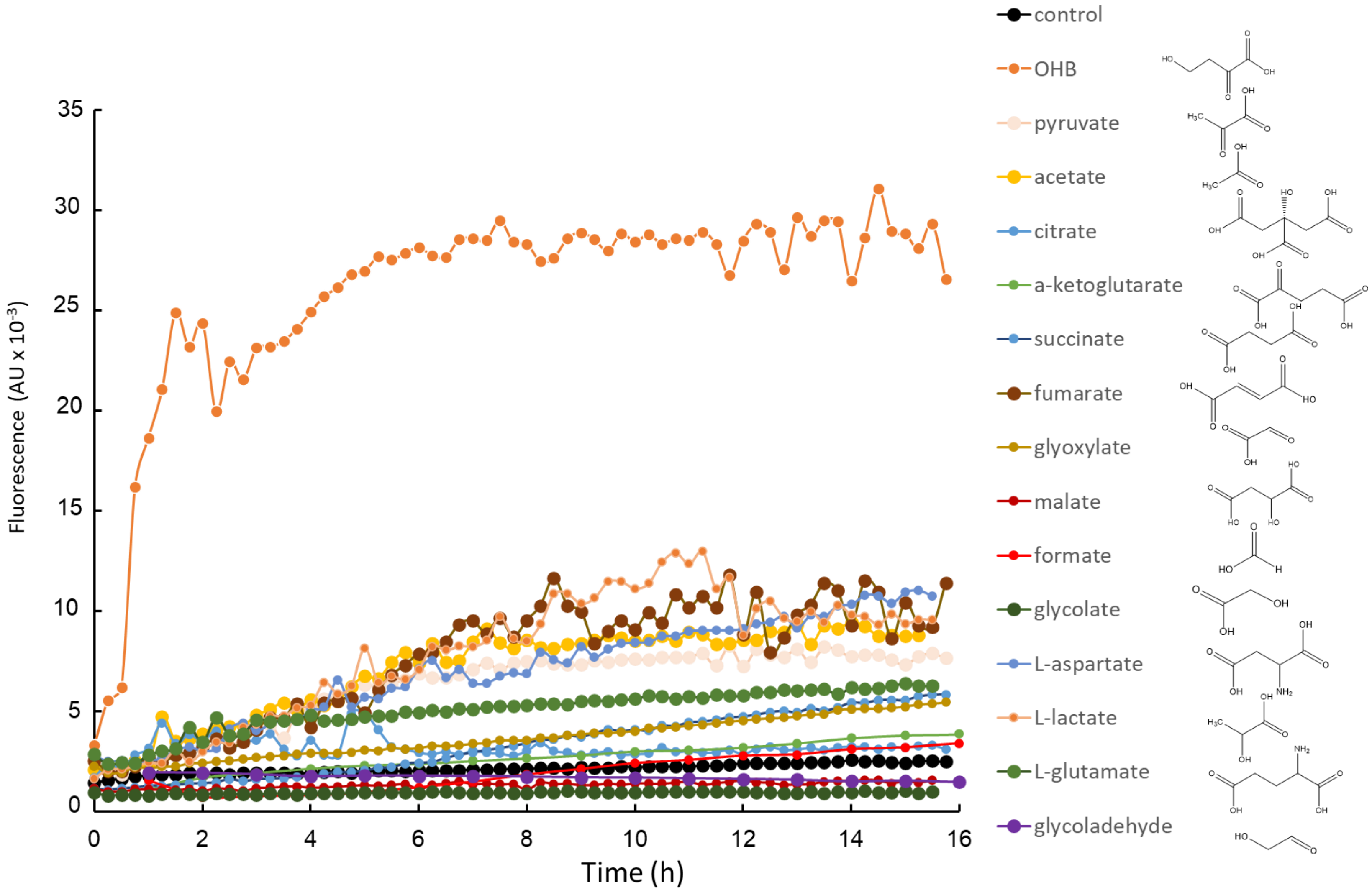

Figure S5: Kinetic of fluorescence response of XynR-based biosensor to various metabolites. The strain MGΔ7 transformed with pBS3 was incubated in the presence of various metabolites added at 10 mM except OHB which was 1 mM). The fluorescence was monitored over time from three independent replicates. Data reported are the mean of the three biological replicates and SD has been removed to simplify the visualisation of the figure but overall, the CV for each curve was in the range of 10 %

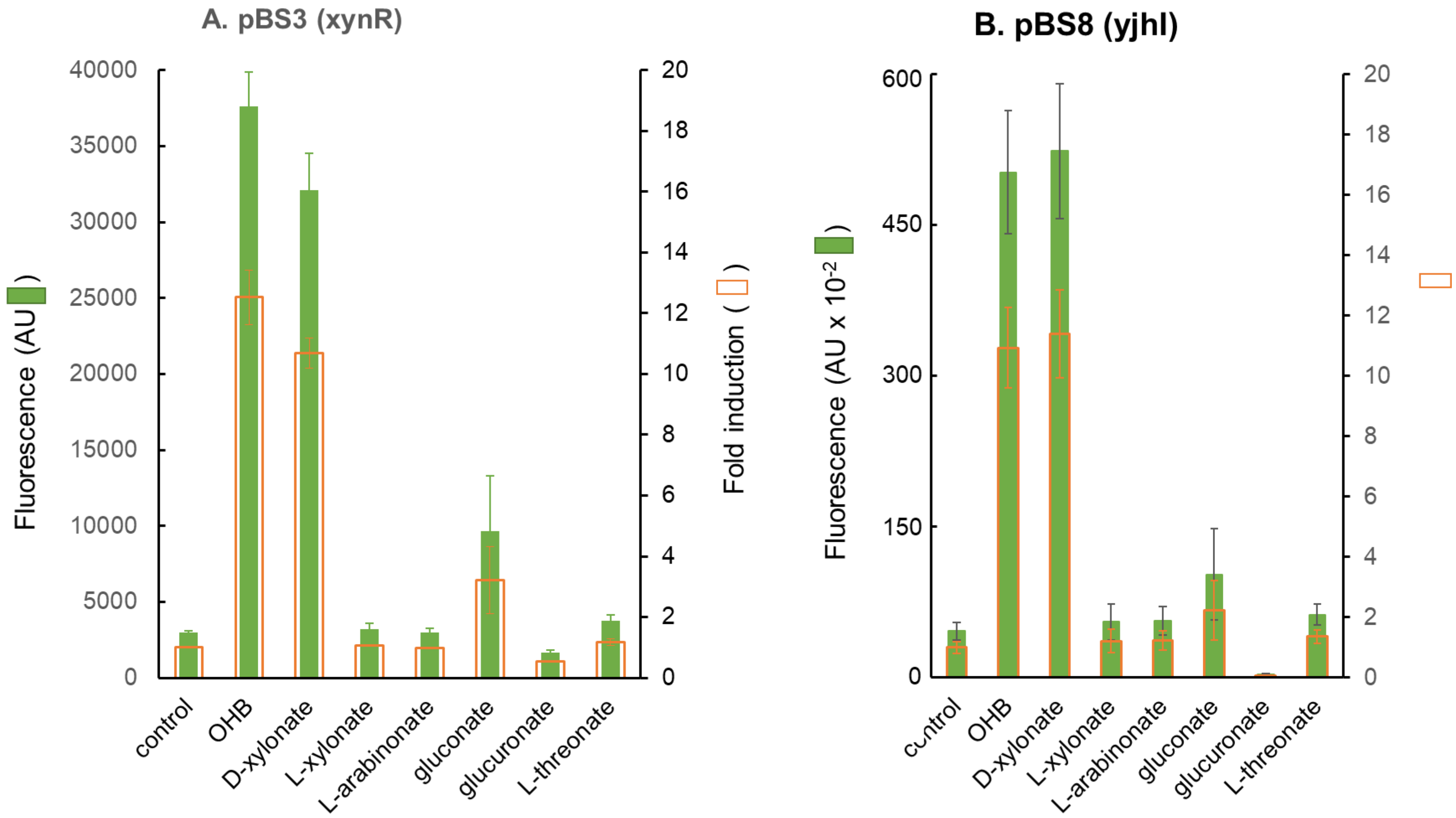

Figure S6: Effect of sugar acids on Xynr and yjhI-based biosensor. To the culture of strain MGΔ7 transformed with pBS3 (A) or pBS8 (B) was added various sugar acids at 20 mM. OHB (1 mM) and D-xylonate (20 mM) were used as control. Data are the mean ± SD (shown by bar on the histogram) of three biological replicates.

A

name	Ori	structure of promoter of detector module
pBS_JF1 (= pBS3)	p15A	P_{xynr} : RBS_{xynR}: xynR
pBS_JF2	p15A	P_{xynr} : RBS_{01}: xynR
pBS_JF3	p15A	P_{J23119}: RBS_{01}: xynR
pBS_JF4	p15A	P_{J23100}: RBS_{01}: xynR
pBS_JF5	p15A	P_{J23104}: RBS_{01}: xynR
pBS_JF6	p15A	P_{J23106}: RBS_{1}: xynR

B

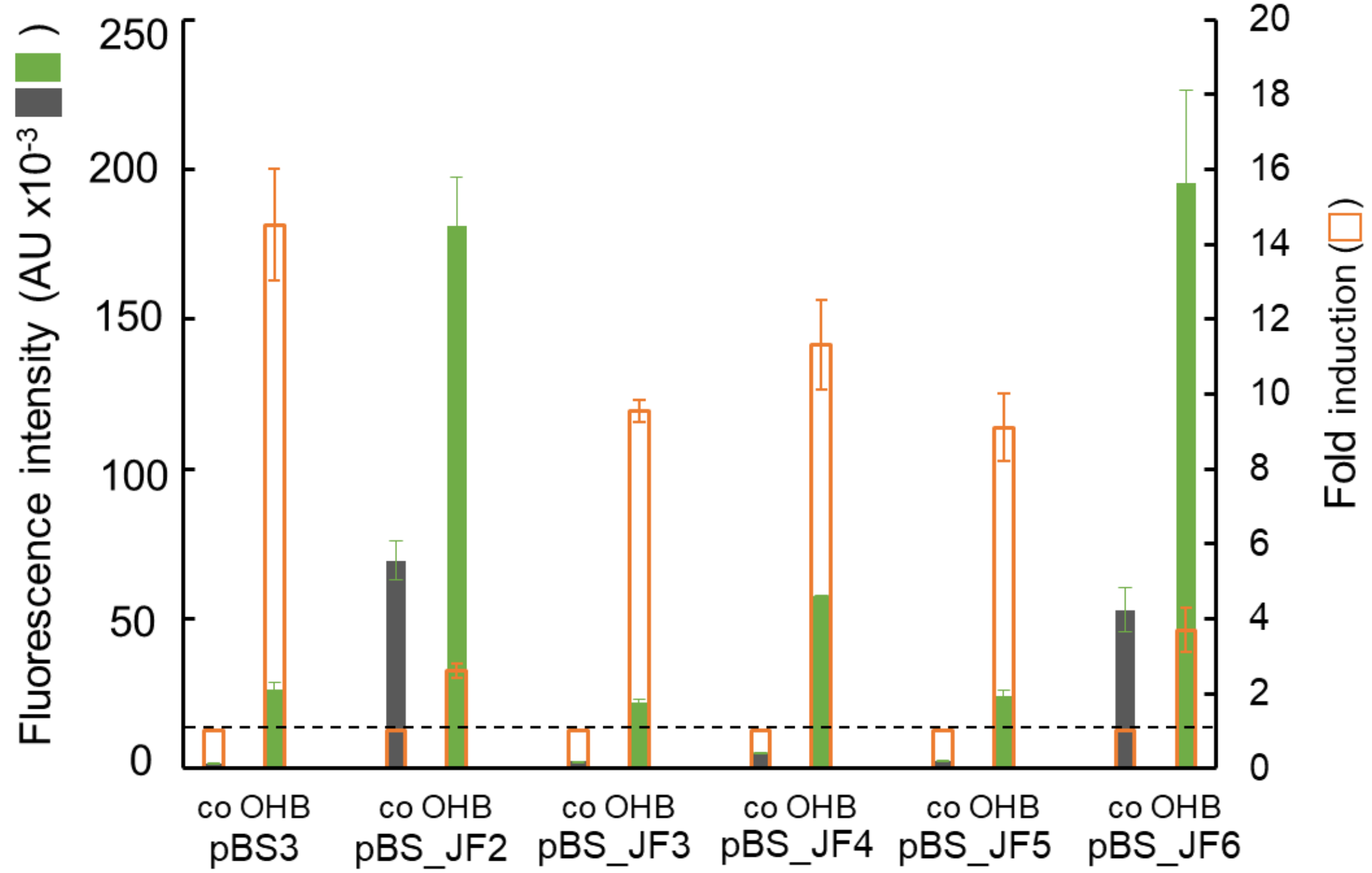

Figure S7: Effect of promoter strength on the dynamic response of the XynR-based biosensor to D-xylonate and OHB. In (**A**) is reported the structure of the XynR promoter and the name of the corresponding medium copy plasmid. In (**B**) is provided the fluorescence of MGΔ7 strain transformed by the different genetic constructs after 12 h incubation with 1 mM OHB. (**C**) is the same as (**B**) except that KDX was used at 1 mM. Data are the mean ± SD (shown by bars on the histogram) of three biological replicates.

References cited

Alkim C, Farias D, Fredonnet J, Serrano-Bataille H, Herviou P, Picot M, Slama N, Dejean S, Morin N, Enjalbert B, Francois JM (2022) Toxic effect and inability of L-homoserine to be a nitrogen source for growth of Escherichia coli resolved by a combination of in vivo evolution engineering and omics analyses. Front Microbiol 13:1051425 doi:10.3389/fmicb.2022.1051425

Baba T, Ara T, Hasegawa M, Takai Y, Okumura Y, Baba M, Datsenko KA, Tomita M, Wanner BL, Mori H (2006) Construction of Escherichia coli K-12 in-frame, single-gene knockout mutants: the Keio collection. Mol Syst Biol 2:2006 0008 doi:10.1038/msb4100050

Cherepanov PP, Wackernagel W (1995) Gene disruption in Escherichia coli: TcR and KmR cassettes with the option of Flp-catalyzed excision of the antibiotic-resistance determinant. Gene 158(1):9-14

Frazao CR, Maton V, Francois JM, Walther T (2018) Development of a Metabolite Sensor for High-Throughput Detection of Aldehydes in Escherichia Coli. Front Bioeng Biotechnol 6:118 doi:10.3389/fbioe.2018.00118

Walther T, Calvayrac F, Malbert Y, Alkim C, Dressaire C, Cordier H, Francois JM (2018) Construction of a synthetic metabolic pathway for the production of 2,4-dihydroxybutyric acid from homoserine. Metab Eng 45:237-245 doi:10.1016/j.ymben.2017.12.005
